# Circular non-coding RNA *ANRIL* modulates ribosomal RNA maturation and atherosclerosis in humans

**DOI:** 10.1038/ncomms12429

**Published:** 2016-08-19

**Authors:** Lesca M. Holdt, Anika Stahringer, Kristina Sass, Garwin Pichler, Nils A. Kulak, Wolfgang Wilfert, Alexander Kohlmaier, Andreas Herbst, Bernd H. Northoff, Alexandros Nicolaou, Gabor Gäbel, Frank Beutner, Markus Scholz, Joachim Thiery, Kiran Musunuru, Knut Krohn, Matthias Mann, Daniel Teupser

**Affiliations:** 1 Institute of Laboratory Medicine, Ludwig-Maximilians-University Munich, 81337 Munich, Germany; 2 LIFE—Leipzig Research Center for Civilization Diseases, Universität Leipzig, 04103 Leipzig, Germany; 3 Department of Proteomics and Signal Transduction, Max Planck Institute of Biochemistry, 82152 Martinsried, Germany; 4 Department of Vascular and Endovascular Surgery, Ludwig-Maximilians-University Munich, 81337 Munich, Germany; 5 Institute of Laboratory Medicine, Clinical Chemistry and Molecular Diagnostics, University Hospital Leipzig, 04103 Leipzig, Germany; 6 Institute for Medical Informatics, Statistics and Epidemiology, University Leipzig, 04107 Leipzig, Germany; 7 Department of Medicine, Perelman School of Medicine at the University of Pennsylvania, Philadelphia, Pennsylvania 19104, USA; 8 Department of Genetics, Perelman School of Medicine at the University of Pennsylvania, Philadelphia, Pennsylvania 19104, USA; 9 Interdisciplinary Center for Clinical Research, University Leipzig, 04103 Leipzig, Germany

## Abstract

Circular RNAs (circRNAs) are broadly expressed in eukaryotic cells, but their molecular mechanism in human disease remains obscure. Here we show that *circular antisense non-coding RNA in the INK4 locus* (*circANRIL*), which is transcribed at a locus of atherosclerotic cardiovascular disease on chromosome 9p21, confers atheroprotection by controlling ribosomal RNA (rRNA) maturation and modulating pathways of atherogenesis. *CircANRIL* binds to pescadillo homologue 1 (PES1), an essential 60S-preribosomal assembly factor, thereby impairing exonuclease-mediated pre-rRNA processing and ribosome biogenesis in vascular smooth muscle cells and macrophages. As a consequence, *circANRIL* induces nucleolar stress and p53 activation, resulting in the induction of apoptosis and inhibition of proliferation, which are key cell functions in atherosclerosis. Collectively, these findings identify *circANRIL* as a prototype of a circRNA regulating ribosome biogenesis and conferring atheroprotection, thereby showing that circularization of long non-coding RNAs may alter RNA function and protect from human disease.

## Abstract (web summary)

Circular RNAs are widely expressed in eukaryotic cells but their functions and mechanisms of action are still being elucidated. Here the authors show that *circANRIL* modulates rRNA maturation and confers protection again atherosclerosis.

## Graphical abstract

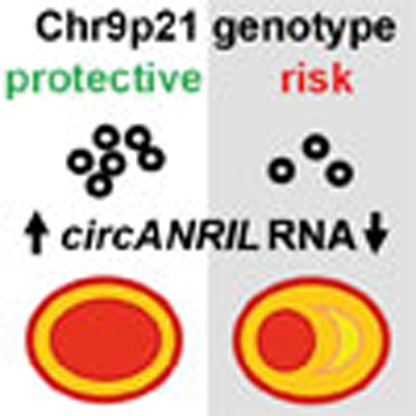

Deep sequencing combined with novel bioinformatics approaches led to the discovery that a significant portion of the human transcriptome is spliced into RNA loops^1^,^2^,^3^. These circular RNAs (circRNAs) do not retain the exon order defined by their genomic sequence and are thought to originate from non-canonical splicing of a 5′ splice site to an upstream 3′ splice site^4^. Recent studies suggest that exon circularization may depend, in part, on inverted repeats or flanking intronic complementary sequences^5^,^6^ but little is known about the functions of these highly stable RNA forms.

Before the finding that circRNAs are abundantly transcribed in humans, there were few reports of circRNAs in mammals. One of the earliest examples is the *sex determining region of Chr Y* (*Sry*) gene in mice, the Y chromosome encoded master regulator of the testis-determining pathway^7^. *Sry* may be expressed as circular and linear transcripts and circularization is thought to be a mechanism to escape translation^7^,^8^. *Sry* was also shown to serve as a competing endogenous RNA of miRNA-138 (ref. 9), and a similar ‘miRNA sponging' function has been demonstrated for a transcript antisense to *cerebellar degeneration related protein 1* (*CDR1as/ciRS-7*)^2^,^9^. *CDR1as* contains ∼70 binding sites for miR-7 and acts to suppress miR-7 activity, resulting in increased levels of miR-7 target genes and functions^2^,^9^. However, only few circRNAs harbour multiple binding sites for miRNAs^10^, suggesting that these abundant RNAs may have other unknown regulatory functions.

Previous work indicated that the long non-coding RNA (lncRNA) *ANRIL*(*CDKN2B-AS1*), which is transcribed at the cardiovascular disease risk locus on chromosome 9p21 (refs 11, 12, 13), is capable of forming RNA circles^14^, yet the functional relevance in human health and disease is unknown. *ANRIL* is differentially expressed by the genotype at 9p21 (for review see ref. 15) and increased linear *ANRIL* (*linANRIL*) was associated with increased atherosclerosis^16^. Recent studies suggest an important role for Alu elements in epigenetic gene regulation by *linANRIL*^17^. These Alu repeats and distal *ANRIL* exons are not conserved in non-primate species^18^, suggesting a primate-specific gain of function of this lncRNA. Here we identify a molecular effector mechanism of circular *ANRIL* (*circANRIL)* using proteomic screening, bioinformatics and functional studies. We demonstrate that *circANRIL* regulates the maturation of precursor ribosomal RNA (pre-rRNA), thus controlling ribosome biogenesis and nucleolar stress. In concert, *circANRIL* confers disease protection by modulating apoptosis and proliferation in human vascular cells and tissues, which are key cellular functions in atherosclerosis.

## Results

### Association of *circANRIL* with atheroprotection at human 9p21

**Figure 1:**
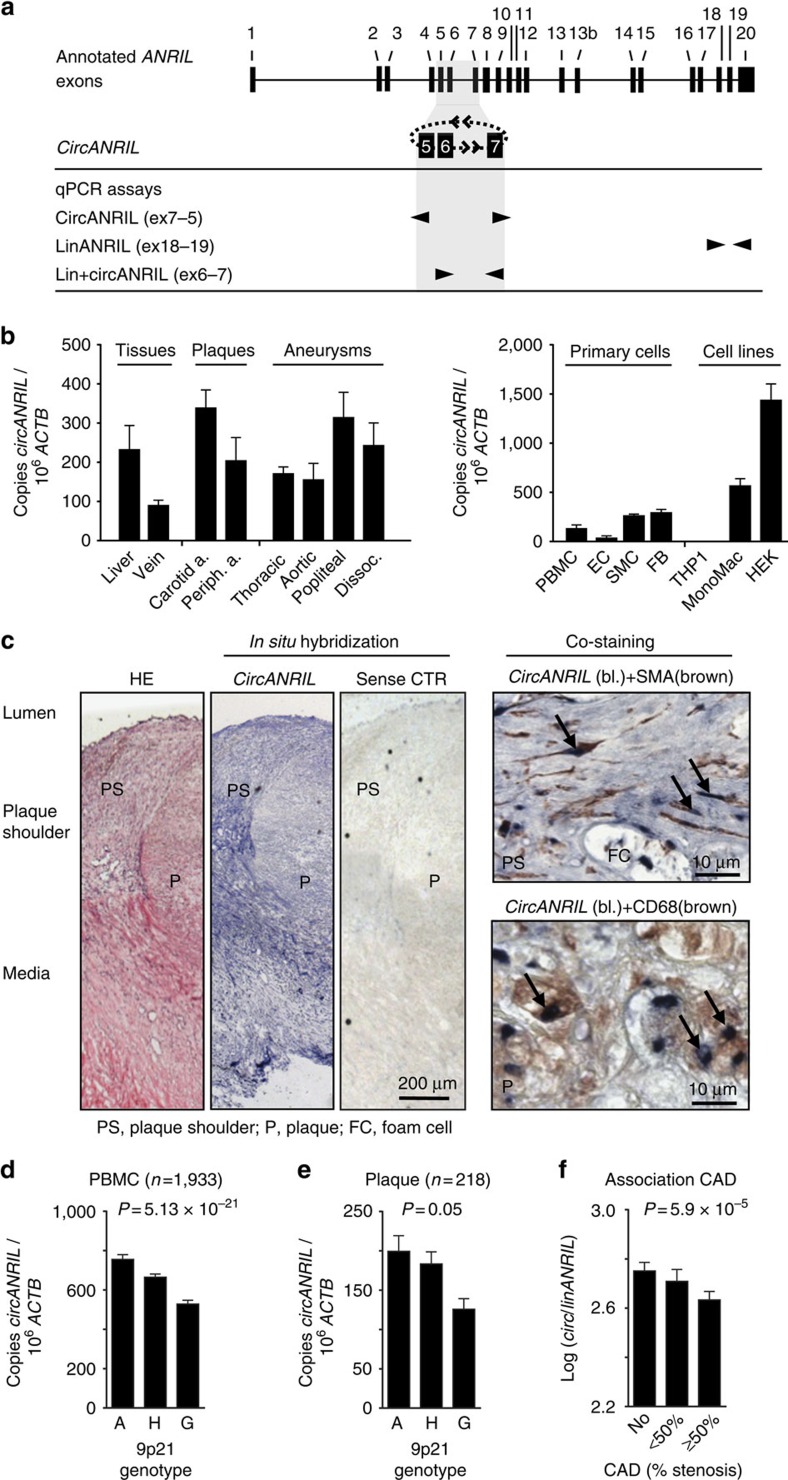
*CircANRIL* expression in human vascular tissue and association with atheroprotection at 9p21. (**a**) Schematic of *circANRIL* at 9p21 and qPCR assays for isoform specific quantification. *CircANRIL* contains exons 5, 6 and 7. Exon 7 is non-canonically spliced to exon 5. (**b**) qPCR analysis of *circANRIL* expression in human tissues, primary cells and cell lines. Beta actin (*ACTB*), house-keeping gene; FB, adventitial fibroblasts; HEK, HEK-293 cell line; THP1 and MonoMac, human monocytic cell line. Analyses were done in RNA pools of at least three donors. (**c**) *In situ* hybridization of *circANRIL* in human atherosclerotic plaque and co-localization with smooth muscle actin (SMA)-positive cells and macrophages (CD68). Sense control (CTR)-negative control. Representative staining out of three biological replicates. (**d**) Association of *circANRIL* with 9p21 haplotypes in PBMC from CAD patients. Protective (A, *n*=498), heterozygote (H, *n*=979) and risk (G, *n*=456) haplotypes. (**e**) Association of *circANRIL* with 9p21 haplotypes A (*n*=49), H (*n*=114) and G (*n*=55) in endarterectomy specimens. (**f**) Association of *circ/linANRIL* ratio in PBMC with severity of CAD (No, *n*=745; <50% stenosis, *n*=392; ≥50% stenosis, *n*=747). Data are given as mean±s.e.m., and associations were calculated using robust linear regression models.

We systematically investigated the exon structure of *circANRIL* in human cell lines and primary cells (Fig. 1a and Supplementary Fig. 1). Using outward-facing primers and PCR analysis of reverse-transcribed RNA, we observed several species of *circANRIL* isoforms. The predominant *circANRIL* isoform consisted of exons 5, 6 and 7, where exon 7 was non-canonically spliced to exon 5 (Fig. 1a and Supplementary Fig. 1). We focused on this isoform for detailed functional characterization and further refer to it as *circANRIL*. *CircANRIL* was expressed in both healthy and diseased human vascular tissues, as well as smooth muscle cells (SMC) and monocyte/macrophages (Fig. 1b), which play an important role in atherogenesis. *CircANRIL* levels were relatively low compared with abundant housekeeping mRNAs such as actin beta (*ACTB*; Fig. 1b), but comparable to the levels of other human circular RNAs such as the previously described *CDR1as*^2^ or *circular* *HPRT1* (*circHPRT1*), which was identified in the present study (Supplementary Fig. 2a). Nevertheless, *circANRIL* RNA levels were on average 9.7-fold higher than levels of *linANRIL* RNA when we analysed a panel of different human cell types and tissues (Supplementary Fig. 2a,b). *CircANRIL* was also more stable than *linANRIL* (Supplementary Fig. 3). The latter is in line with previous reports on other circular RNAs^1^,^19^. To determine the spatial distribution of *circANRIL* expression in the context of vascular atherogenesis, we performed RNA *in situ* hybridization using a *circANRIL*-specific probe (Fig. 1c and Supplementary Fig. 4). We detected *circANRIL* in SMC and in CD68-positive macrophages in human atherosclerotic plaques (Fig. 1c).

We next tested for an association of *circANRIL* expression with the 9p21 genotype in a large cohort of patients with different burden of coronary artery disease (CAD), as assessed by coronary angiography^17^,^20^. Carriers of the CAD-protective haplotype at 9p21 showed significantly increased expression of *circANRIL* in peripheral blood mononuclear cells (PBMC, *n*=1,933; 14% per protective A-allele; Fig. 1d) and whole blood (*n*=970; *P*=1.86 × 10^−7^; 27% per allele; associations were calculated using robust linear regression models). Differential expression was replicated in endarterectomy specimens, where each protective allele was associated with 13% higher *circANRIL* expression (Fig. 1e). Importantly, the direction of effects for *circANRIL* was inverse to the published results for *linANRIL*, which was downregulated in patients carrying the 9p21 protective genotype^16^,^17^. Consistent with these findings, *circANRIL* expression was inversely correlated with *linANRIL* expression in PBMC of the CAD cohort (*r*=−0.24; *P*=3.72 × 10^−29^; linear regression). We then tested for an association of *circANRIL* with CAD burden. Patients with high *circANRIL* expression developed less CAD (*P*=0.047; odds ratio=0.8; linear regression) and highest *circANRIL*/*linANRIL* ratios were found in patients free of CAD (*P*=5.9 × 10^−5^; odds ratio=0.75; Fig. 1f; linear regression). Calculated by a Mendelian randomization approach, the *circANRIL*/*linANRIL* ratio explained a significant part of the observed association of 9p21 with CAD (*P*=0.002; linear regression). This establishes a causal relationship between the genotype at 9p21, high *circANRIL* expression and CAD protection. Taken together, these data implied an atheroprotective role of *circANRIL* at 9p21, which was inverse to the previously reported proatherogenic function of its linear counterparts^17^.

### *CircANRIL* controls apoptosis and cell proliferation *in vitro*

**Figure 2:**
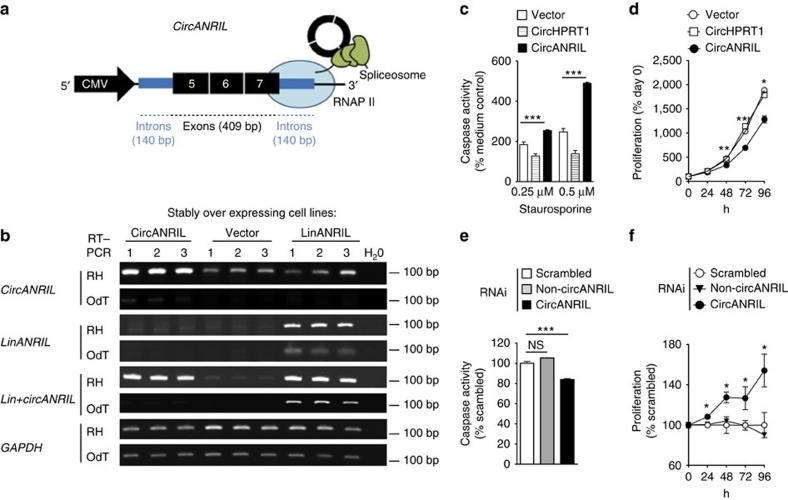
Atherosclerosis-related cell functions in c*ircANRIL*-overexpressing cells. (**a**) Schematic of the vector construct for *circANRIL* stable and transient overexpression. RNA polymerase II (RNAP II). (**b**) *ANRIL* isoform expression in HEK-293 cells that stably express *circANRIL*, *linANRIL* or empty vector (three biological replicates each). PCR of reverse-transcribed RNA, glyceraldehyde 3-phosphate dehydrogenase (*GAPDH*), house-keeping gene; OdT, oligo(d)T primers; RH, random hexamer primers. (**c**) Apoptosis and (**d**) proliferation in HEK-293 cell line stably overexpressing *circANRIL* (pool of 3 biological replicates, and 8 and 12 technical replicates per condition, respectively), compared with overexpression of an unrelated circular RNA (*circHPRT1*). Apoptosis (**e**) and proliferation (**f**) after RNAi against *circANRIL* or non-*circANRIL* or scrambled (SCR) siRNA control. Quadruplicate measurements per condition. *P<0.05; **P<0.01; ***P<0.001. Comparison of multiple groups was done using analysis of variance, and Tukey was performed as *post hoc* test in **c**,**e**. Two-tailed unpaired Student's *t*-test in **d**,**f**. Data are given as mean±s.e.m.

We next wanted to test whether *circANRIL* was biologically active and controlled cell functions related to atherosclerosis. To mimic increased levels of *circANRIL* RNA, as observed in patients that were protected from atherosclerosis, we set out to generate cell lines stably overexpressing the *circANRIL* RNA consisting of exons 5, 6 and 7. Since circularization of *ANRIL* was spliceosome-dependent (Supplementary Fig. 5), we predicted that flanking intronic sequences might be relevant for circularization and constructed a vector, where exonic sequences were surrounded by 140 bp intronic sequences 5′ of exon 5 and 3′ of exon 7 (Fig. 2a and Supplementary Table 1). On the basis of this approach we succeeded to express *circANRIL* RNA in 12 of 30 (40%) stably transfected HEK-293 cells, which was validated by *circANRIL*-specific quantitative reverse transcription PCR (qPCR) (Fig. 2b). On average, stably overexpressing circANRIL cells had 16-fold higher *circANRIL* expression levels compared with endogenous *circANRIL.* For functional studies, we selected cell lines with a modest 3-fold overexpression to resemble a more physiological situation (Fig. 2c–f). Corroborating our previous results, measuring *circANRIL* RNA turnover rates following inhibition of RNA polymerase II transcription with actinomycin D, the overexpressed *circANRIL* was more stable than *linANRIL* (Supplementary Fig. 6).

Studies of key cell functions of atherogenesis in *circANRIL*-overexpressing cells revealed increased apoptosis (Fig. 2c) and decreased proliferation (Fig. 2d). Overexpression of the unrelated circular RNA *circHPRT1* (Supplementary Fig. 7 and Supplementary Table 1) did not trigger these effects (Fig. 2c,d), ruling out that proapoptotic and antiproliferative functions are generic roles of all circular RNAs. In further support for the specificity of the observed consequences of *circANRIL* overexpression, effects could be reversed by small interfering RNA (siRNA)-mediated downregulation of *circANRIL* RNA, but not by using *linANRIL-*specific or scrambled siRNA controls (Fig. 2e,f and Supplementary Fig. 8).

**Figure 3:**
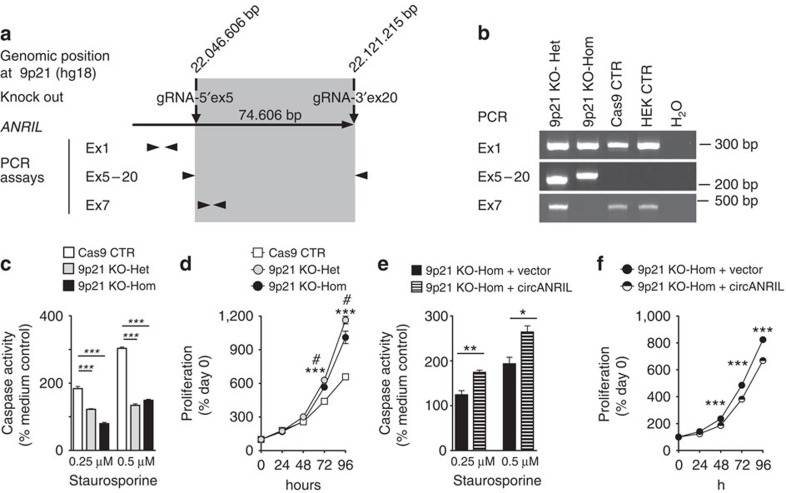
Role of endogenous *circANRIL* on apoptosis and cell proliferation. (**a**) Schematic of CRISPR/Cas9-mediated knockout of *ANRIL* in HEK-293 cells. Genomic regions encompassing exons (Ex) 5–20 were deleted. Arrowheads indicate location and orientation of primers used for genotyping. (**b**) PCR genotyping. Sequencing of PCR products from Ex5–20 assay validated successful deletion in mutant cell lines (Supplementary Table 2). (**c**) Apoptosis and (**d**) proliferation in heterozygous and homozygous *ANRIL* knockout or control cell lines. As control, Cas9 was overexpressed without guide RNAs (gRNAs). (**e**) Apoptosis and (**f**) proliferation in homozygous knockout cells following transient re-expression of *circANRIL*. *P<0.05; **P<0.01; ***P<0.001. (**d**) Heterozygous (***P<0.001) or homozygous (^#^*P*<0.05) *ANRIL* knockout versus control cell lines, respectively. Comparison of multiple groups was done using analysis of variance, and Tukey was performed as *post hoc* test in **c**. Two-tailed unpaired Student's *t*-tests were applied in **d**–**f**. Data are given as mean±s.e.m.

We next deleted exons 5–20 from the *ANRIL* locus in HEK-293 cells using CRISPR/Cas9 technology and established heterozygous and homozygous knockout cell lines (Fig. 3a,b and Supplementary Table 2). Deletion of exons 5–20 impairs *linANRIL* RNA isoforms, which contain at least exons 5 and 6 (ref. 17). The deletion also affects several *circANRIL* isoforms, including the major isoform consisting of exons 5–7, which we are studying. Our analysis revealed that loss of *ANRIL* conferred a gene dosage-dependent reduction in apoptotic rate (Fig. 3c) and led to increased cellular proliferation (Fig. 3d). We stress that increased proliferation and reduced apoptosis cannot solely be ascribed to either deletion of *linANRIL* RNAs nor to deletion of circRNAs. For this reason, using an expression vector to restore *circANRIL* but not *linANRIL* expression in these cell lines, we re-expressed the major *circANRIL* isoform consisting of exons 5–7 in the knockout cell line that misses exons 5–20. We showed that presence of *circANRIL* alone was sufficient to increase cell apoptosis (Fig. 3e) and to reduce proliferation (Fig. 3f) indicating that *circANRIL* mediated its effects likely independent of *linANRIL* at 9p21. Together, this genetic analysis demonstrates that *circANRIL* is a physiologically relevant modulator of apoptosis and proliferative capacity.

### *CircANRIL* acts independent of *CDKN2A/B* and of miRNA sponging

**Figure 4:**
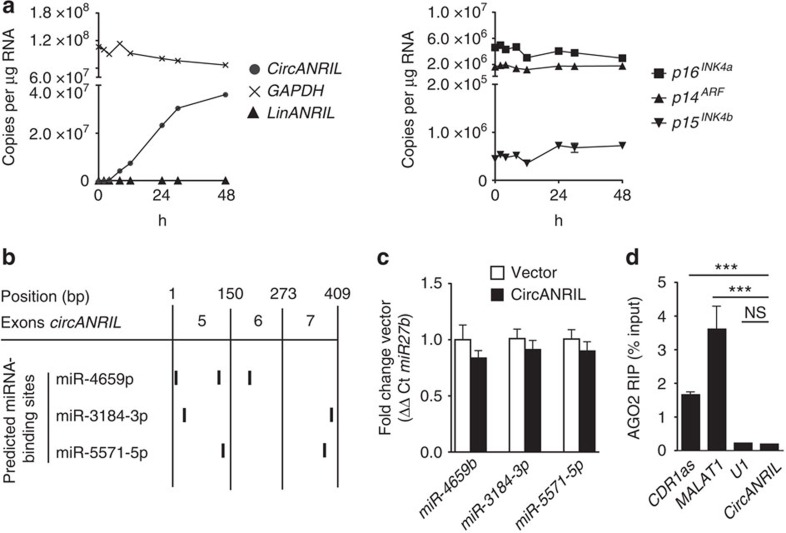
*CircANRIL* does not regulate 9p21 protein-coding genes and lacks miRNA sponging activity. (**a**) Overexpression of *circANRIL* in HEK-293 cells does not modulate endogenous *linANRIL* RNA abundance as measured by qPCR (left panel). Expression of *p16*^*INK4a*^ and *p14*^*ARF*^, encoded by *CDKN2A*, and *p15*^*INK4b*^, encoded by *CDKN2B*, in *circANRIL*-overexpressing cells (right panel) (pool of three biological replicates, quadruplicate qPCR measurements). (**b**) Prediction of miRNA-binding sites in *circANRIL* using miRanda and TargetSpy algorithms. (**c**) miRNA expression normalized to miR-27b as measured by qPCR (four biological replicates, quadruplicate measurements). (**d**) RNA immunoprecipitation (RIP) of AGO2. Analysis of precipitated RNAs: *CDR1as*, *MALAT1*, positive controls; *U1*, negative control (RIP was performed in a pool of three biological replicates, quadruplicate qPCR measurements). ***P<0.001. NS, not significantly different. (analysis of variance, and Tukey as *post hoc* test). Data are given as mean±s.e.m.

Previous work suggested that *ANRIL* regulated the expression of the tumour suppressor genes *cyclin-dependent kinase inhibitors A and B* (*CDKN2A* and *B*)^21^,^22^, located adjacent to *ANRIL*'s genomic position. We therefore investigated whether *circANRIL* affected the expression of these genes, but we detected no significant changes (Fig. 4a). Furthermore, we observed that *linANRIL* remained unaffected by overexpressing *circANRIL* (Fig. 4a), excluding *cis*-regulation of 9p21 transcripts as molecular effector mechanism. Other work has shown that circRNAs, which were enriched for miRNA seeds, may serve as miRNA sponges^2^,^9^. However, we only identified a maximum of three binding sites for miR-4659a/b, miR-3184-3p and miR-5571-5p (Fig. 4b) in *circANRIL*. Moreover, expression of these miRNAs was not changed in *circANRIL*-overexpressing cells (Fig. 4c), and genome-wide expression arrays in *circANRIL* and control cells did not support an induction of miRNA-regulated networks (Supplementary Fig. 9; Gene Expression Omnibus GSE65392). In addition, *circANRIL* did not bind to argonaute 2 (AGO2; Fig. 4d) of the RNA-induced silencing complex, which is responsible for miRNA-dependent degradation of target RNAs. These data suggested that *circANRIL* controlled atheroprotective cell functions through a novel molecular mechanism, independent of *cis*-regulation at 9p21 and miRNA sponging.

### *CircANRIL* is a component of a pre-ribosomal assembly complex

**Figure 5:**
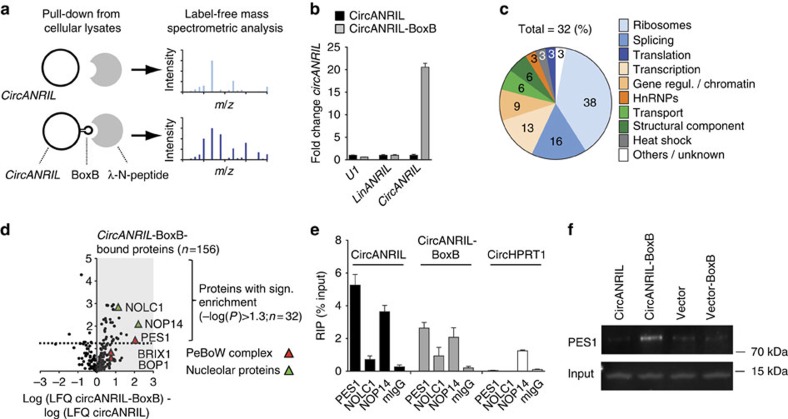
Identification of *circANRIL-*binding proteins. (**a**) Schematic of λN-peptide-mediated capture of *circANRIL*-BoxB from cellular lysates of stably overexpressing HEK-293 cell lines, and label-free mass spectrometric quantification. Experiments were performed in a pool of three biological replicates (quadruplicate measurements). (**b**) Quantification of RNAs by qPCR after λN-peptide capture (quadruplicate measurements). (**c**) Summary of *circANRIL*-BoxB-bound proteins according to annotated (KEGG, GO) and published functions. (**d**) Volcano plot of label-free quantification (LFQ) of *circANRIL*-BoxB-bound proteins compared with *circANRIL* input control. (**e**) RIP of PES1, NOLC1, NOP14 and mouse IgG (mIgG) control (RIP was performed in a pool of three biological replicates, quadruplicate measurements). (**f**) Western blot of PES1 after λN-peptide-mediated circANRIL-BoxB capture using nuclear extracts from indicated cell lines.

Since *linANRIL* has protein-binding capacity^17^,^21^,^22^, we performed a proteomic screen to identify potential *circANRIL*-binding proteins (Fig. 5). To this end, we generated HEK-293 cell lines with stable overexpression of *circANRIL* engineered to contain RNA hairpin BoxB-sequences^23^ (*circANRIL*-BoxB; Supplementary Fig. 10 and Supplementary Table 1). This allowed capture of *circANRIL*-bound proteins in cellular lysates via high affinity interaction of the BoxB RNA hairpin with bacteriophage λ transcriptional antiterminator protein N (λN-peptide) coupled to beads (Fig. 5a–d). Circularization of *circANRIL* was not impaired by the insertion of *BoxB*-sequences (Supplementary Fig. 10). As controls for the capture, we used protein extracts from HEK-293 cell lines with stable overexpression of *circANRIL* without BoxB RNA hairpin sequences (Fig. 5a). On the basis of quantitative reverse transcription PCR analysis, we validated that *circANRIL* was highly enriched following bead-mediated capture (Fig. 5b), demonstrating that our isolation strategy was specific and selective. Capture experiments were performed in quadruplicates and we used 2 μg of precipitated protein from each of the two conditions for label-free mass spectrometric analyses. Here we detected 32 proteins with significant enrichment in extracts from *circANRIL*-BoxB-overexpressing cells compared with extracts from control cells (Fig. 5c,d and Supplementary Table 3). The majority of identified proteins was involved in ribosome biogenesis and assembly (38%) and regulation of RNA splicing (16%) (Fig. 5c) suggesting that *circANRIL* may play a role in these important cellular processes. Strongest binding was determined for nucleolar protein 14 (NOP14, Fig. 5d), which is involved in the formation of the nucleolar 40S ribosomal subunit^24^, and for pescadillo zebrafish homologue 1 (PES1, Fig. 5d), which is a component of the PES1–BOP1–WDR12 (PeBoW) complex. This complex is homologous to yeast Nop7-Erb1-Ymt1 (refs 25, 26) and a key regulator of large 60S ribosome subunit biogenesis^27^,^28^. The PeBoW complex assembles with pre-ribosomes and binds to precursor rRNA (pre-rRNA), thereby facilitating the processing of 47S pre-rRNA to mature 28S and 5.8S rRNA by exonucleases^25^,^29^,^30^. Block of proliferation 1 (BOP1), which was demonstrated to bind PES1 (refs 26, 30), was also present in *circANRIL*-BoxB extracts, as well as ribosome biogenesis protein BRX1 homologue (BRIX1) and nucleolar and coiled-body phosphoprotein 1 (NOLC1), two other PeBoW complex-associated proteins^27^ (Fig. 5d).

To corroborate our mass spectrometric analysis by an independent method, we chose to analyse *circANRIL*–protein interactions by RNA immunoprecipitation (RIP). qPCR analysis after RIP revealed the interaction of *circANRIL* RNA with the PeBoW complex members PES1 and NOP14, where *circANRIL* binding to PES1 was the strongest interaction tested (Fig. 5e). PES1 binding was not detected for circularized *HPRT1* (*circHPRT1*), indicating that the binding of *circANRIL* to PES1 was not due to a generic interaction of PES1 with all species of circular RNAs in a cell (Fig. 5e). λN-peptide-mediated pull-down of *circANRIL-*BoxB from nuclear extracts followed by western blotting validated *circANRIL* binding to PES1 protein (Fig. 5f) but not to NOP14. We therefore focused subsequent functional studies on PES1 as main *circANRIL*-interacting protein.

### *CircANRIL* controls pre-rRNA maturation and nucleolar stress

**Figure 6:**
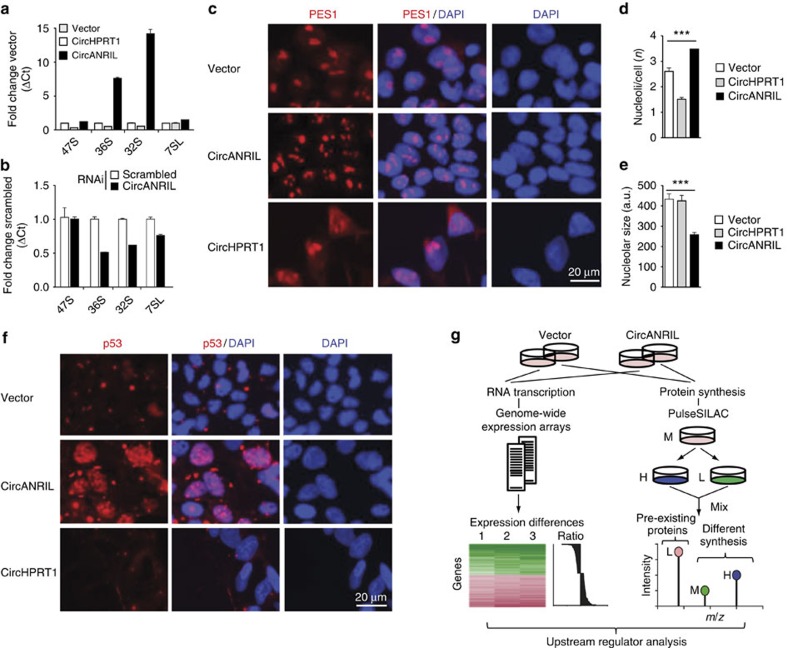
rRNA maturation defects and nucleolar stress in *circANRIL*-overexpressing cells. (**a**) Relative quantification of pre-rRNA in *circANRIL*- or in *circHPRT1-*overexpressing HEK-293 cells using isoform-specific qPCRs (RNA from a pool of three biological replicates, quadruplicate measurements). (**b**) Pre-rRNA levels after RNAi against *circANRIL* or using scrambled siRNA control (quadruplicate measurements per condition). (**c**) Immunofluorescent staining of PES1 in *circANRIL*- or in *circHPRT1*-overexpressing HEK-293 or empty vector control cells. Quantification of (**d**) nucleoli and (**e**) nucleolar size in *circANRIL*- or *circHPRT1*-overexpressing or empty vector control cells (*** *P*<0.001). Data are given as mean±s.e.m. (analysis of variance, and Tukey as *post hoc* test). (**f**) Immunofluorescent staining of p53 (red) in *circANRIL*- or *circHPRT1*-overexpressing or empty vector control cells. (**g**) Schematic of transcriptome and proteome analyses in *circANRIL*-expressing HEK-293 cells and in control cells by genome-wide expression arrays and by pulseSILAC, respectively. For results of pathway analyses and procedure, see Supplementary Tables 4–6 and Methods section, respectively. L/M/H, light/medium/heavy medium.

PES1 is essential for PeBoW complex function, and inhibition of this complex leads to accumulation of premature rRNA isoforms (Supplementary Fig. 11)^29^,^30^. Using isoform-specific qPCRs^31^, we detected a significant accumulation of 36S and 32S pre-rRNA in *circANRIL*-overexpressing HEK-293 cell lines but not in *circHPRT1*-overexpressing cells (Fig. 6a). Expression levels of 47S pre-rRNA and 7SL RNA, which is independently transcribed by RNA polymerase III, were not altered in either cell line (Fig. 6a). Corroborating results from our qPCR analysis, northern blotting revealed an accumulation of 32S and 36S pre-rRNAs in *circANRIL*-overexpressing cells (Supplementary Fig. 12). Effects on pre-rRNAs could be reversed by siRNA against *circANRIL* (Fig. 6b) implying that *circANRIL* negatively regulated PeBoW activity and rRNA maturation. This regulation of ribosome maturation is a previously undescribed function for *circANRIL* and a yet undescribed role for circular RNAs in general.

It is well established that inhibition of rRNA maturation leads to impaired ribosome biogenesis and induction of nucleolar stress^32^, which may be evidenced by small nucleoli, nucleolar disorganization^33^ and p53 stabilization^32^,^34^. Indeed, immunofluorescent stainings of PES1 (Fig. 6c and Supplementary Fig. 13a) and BOP1 (Supplementary Fig. 13b) revealed significantly more (Fig. 6d) and smaller (Fig. 6e) nucleoli in *circANRIL*-overexpressing HEK-293 cells, providing evidence for increased nucleolar stress. Notably, we did not detect an increase in nucleolar number nor a decrease of nucleolar size when we stably overexpressed *circHPRT1* (Fig. 6d,e), providing evidence for the specificity of the observed effects for *circANRIL*. Using immunofluorescent stainings, we also detected a significant accumulation of p53 in the nuclei of *circANRIL*-overexpressing cells (Fig. 6f and Supplementary Fig. 13c). Activation of p53 was further corroborated by proteomic analyses in cell cultures of circANRIL and control cell lines after pulse stable isotope labelling (pulseSILAC), as well as by transcriptome-wide RNA expression analyses (Fig. 6g), which indicated a significant activation of p53 signalling both, at the protein and at the mRNA level (Supplementary Tables 4–6; Gene Expression Omnibus GSE65392).

### *CircANRIL* binding to PES1 prevents rRNA maturation

**Figure 7:**
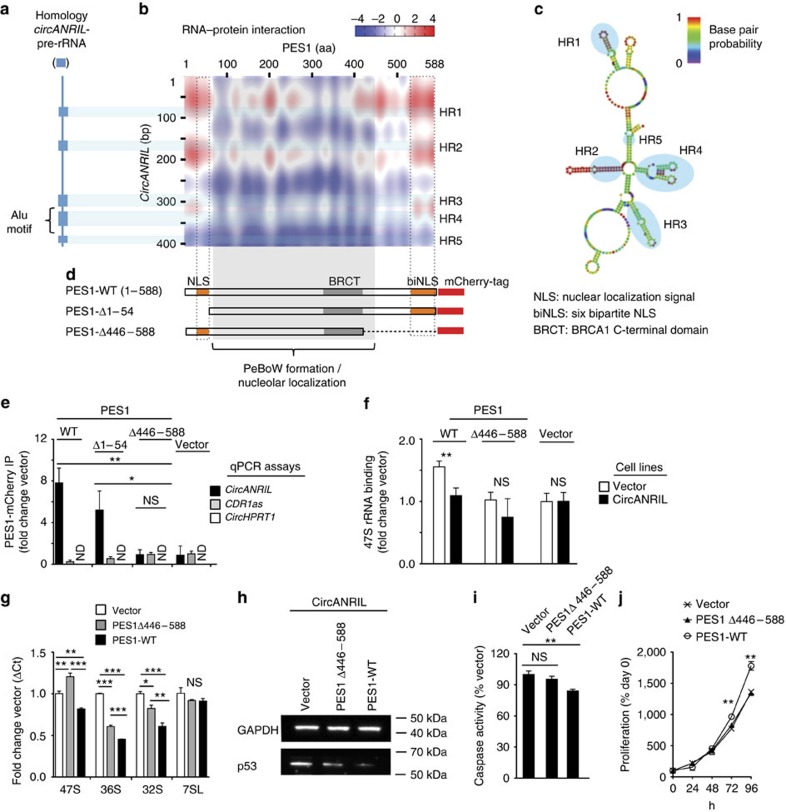
Molecular mechanism of *circANRIL* controlling PES1 function. (**a**) Homology of *circANRIL* and precursor 47S rRNA (blue boxes) determined by BLASTn algorithm. (**b**) Prediction of RNA–protein interaction of *circANRIL* with PES1 using the catRAPID algorithm. Homology regions (HR1–HR5)—predicted RNA–protein interaction motifs in *circANRIL*. (**c**) Secondary structure prediction and HR1–5 of *circANRIL* using the Vienna RNA package^69^. (**d**) Schematic of PES1 with functional protein domains. Wild-type PES1 (PES1-WT) and two mutants lacking the 5′ (PES1Δ1–54) or the 3′ (PES1Δ446–588) lysine-rich NLSs. (**e**) Immunoprecipitation (IP) of PES1 isoforms from HEK-293 cells and quantification of RNA by qPCR (IP was performed in a pool of three biological replicates, quadruplicate measurements). (**f**) Pre-rRNA binding to PES1-WT and PES1Δ446–588 in *circANRIL*-overexpressing and control cells (IP was performed in a pool of three biological replicates, quadruplicate measurements). (**g**) Pre-rRNA and 7SL control RNA in *circANRIL-*expressing HEK-293 cells after transient PES1-WT or PES1Δ446–588 overexpression (pool of three biological replicates, quadruplicate measurements). (**h**) p53 western blot, (**i**), apoptosis and (**j**) cell proliferation in *circANRIL*-overexpressing HEK-293 cells with transient overexpression of PES1-WT or of PES1Δ446–588. Quadruplicate measurements per condition. *P<0.05; **P<0.01; ***P<0.001. NS, not significantly different. Analysis of variance, and Tukey as *post hoc* test in **e**,**g** and **i**. Two-tailed unpaired Student's *t*-tests were applied in **f**,**j**. Data are given mean±s.e.m.

Although *circANRIL* and ribosomal RNA belong to different families of lncRNAs, we observed significant sequence homology (Fig. 7a and Supplementary Table 7). Since *circANRIL* (Fig. 5) and ribosomal RNA both bind to PES1 (ref. 25), we hypothesized that *circANRIL* occupied pre-rRNA-binding sites at PES1, thereby inhibiting exonuclease-mediated processing and maturation of ribosomal RNA. Using two independent algorithms for RNA–protein interaction, *circANRIL*-PES1 binding was predicted to be mediated through RNA domains with high pre-rRNA sequence homology (Fig. 7b and Supplementary Table 7) forming RNA stem loop structures (Fig. 7c), and through the N- and C-terminal domains of PES1 protein (Fig. 7d), respectively. These protein domains are enriched for lysine-rich amino-acid motifs with protein–RNA binding potential^35^ and have structural similarity to nuclear localization signals (NLSs). Previous work has shown that PES1's NLSs are not required for nucleolar localization of PES1 but important for PeBoW complex function^26^,^29^.

To investigate whether *circANRIL* bound to PES1 at the predicted protein domains, we performed protein immunoprecipitation of full-length and truncated PES1 (PES1Δ1-54 or PES1Δ446–588, respectively; Fig. 7d,e, Supplementary Fig. 14 and Supplementary Table 8). We only detected *circANRIL* binding when the C-terminal PES1 protein domain (aa 446–588) was present (Fig. 7e). This domain was also required for 47S and 36S pre-rRNA binding and *circANRIL* prevented pre-rRNA binding to some extent (Fig. 7f and Supplementary Fig. 15). Importantly, full-length but not truncated PES1 restored rRNA maturation in circANRIL-expressing cells (Fig. 7g) and reduced p53 accumulation (Fig. 7h), apoptosis (Fig. 7i) and cellular proliferation (Fig. 7j).

Together, these data establish *circANRIL* as a molecular inhibitor of PES1 by binding at the C-terminal domain of PES1. This may, at least in part, explain the effects of *circANRIL* in rRNA maturation, nucleolar stress and atheroprotective cell functions.

### Translation of *circANRIL* molecular effects to human disease

**Figure 8:**
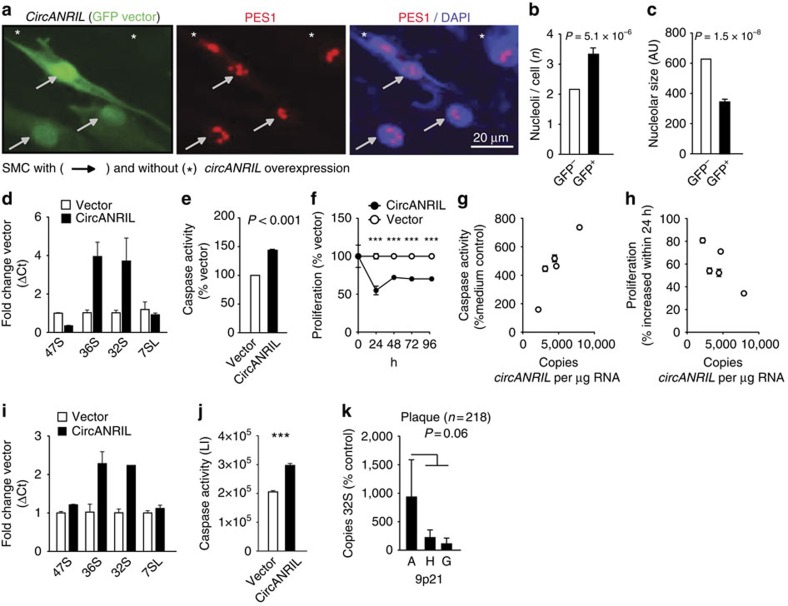
Translation of *circANRIL* molecular mechanisms in human primary cells and human cohorts. (**a**) *CircANRIL* overexpression in primary human SMC using the green fluorescent protein (GFP)-encoding, bicistronic pBI-CMV2 vector (green; Fig. 2a) and immunofluorescent staining of PES1 (red). Quantification of nucleoli (**b**) and nucleolar size (**c**) in *circANRIL*-overexpressing (GFP^+^) SMC and *circANRIL*-negative (GFP^−^) cells. AU, arbitrary units. (**d**) Quantification of pre-rRNAs following *circANRIL* overexpression in SMC. (**e**) Apoptosis and (**f**) proliferation in SMC transfected with *circANRIL*. Quadruplicate measurements per condition. Correlation of endogenous *circANRIL* expression in human SMC with (**g**) apoptosis and (**h**) proliferation (*n*=5; quadruplicate measurements each). (**i**) Quantification of pre-rRNA following *circANRIL* overexpression in iPSC-derived macrophages (Supplementary Fig. 16). (**j**) Apoptosis in iPSC-derived macrophages transfected with *circANRIL*. LI, luminescence intensity. Quadruplicate measurements per condition. (**k**) Association of 32S rRNA expression with 9p21 A (*n*=49), H (*n*=114) and G (*n*=55) haplotypes in endarterectomy specimens. ***P<0.001. Two-tailed unpaired Student's *t*-tests were applied in **b**,**e**,**f** and **j**, and Mann–Whitney *U*-testing in **k**. Data are given mean±s.e.m.

*ANRIL* is evolutionary not conserved between species, and exons 5–7, which are contained in *circANRIL*, are primate-specific^18^. Consequently, no expression of *circANRIL* was detected in mouse tissues using qPCR preventing studies in mouse models. To translate our *in vitro* findings to a physiologically relevant *in vivo* context, we performed functional studies in human primary SMC and human induced pluripotent stem cell (iPSC)-derived macrophages and conducted association studies in human disease cohorts (Fig. 8). *CircANRIL* overexpression in SMC (Fig. 8a–f) increased the numbers of nucleoli (Fig. 8a,b), reduced nucleolar size (Fig. 8a,c), and led to an accumulation of pre-rRNAs (Fig. 8d). Consequently, *circANRIL* induced apoptosis (Fig. 8e) and decreased cell proliferation (Fig. 8f). Using SMC of different patients, we demonstrated that cells with higher endogenous *circANRIL* expression showed a stronger induction of apoptosis and a reduced proliferative capacity as well (Fig. 8g,h). In iPSC-derived macrophages (Supplementary Fig. 16) *circANRIL* overexpression also caused an accumulation of pre-rRNAs (Fig. 8i) and induced apoptosis (Fig. 8j), thereby confirming that *circANRIL*-associated induction of nucleolar stress also occurs in cells that are relevant in atherogenesis *in vivo.*

Further *in vivo* evidence was obtained from human atherosclerotic plaques (*n*=218) (Fig. 8k). Here we observed significant correlations of *circANRIL* with 36S (*r*_s_=0.17, *P*=0.003, Mann–Whitney *U*-test) and 32S pre-rRNA abundance (*r*_s_=0.21, *P*=0.0003; Mann–Whitney *U*-test) but not with 47S pre-rRNA (*r*_s_=−0.01, *P*=not significant; Mann–Whitney *U*-test) or with 7SL control RNA abundance (*r*_s_=0.09, *P*=not significant; Mann–Whitney *U*-test). This indicates that high *circANRIL* levels led to an accumulation of pre-rRNA in human vascular tissue. In this cohort, 32S pre-rRNA was also elevated in patients carrying the CAD-protective allele at 9p21 (Fig. 8k), linking the atheroprotective genotype with high *circANRIL* expression (Fig. 1d–f) and pre-rRNA accumulation *in vivo*. To further translate our findings, we performed pathway analyses of genome-wide expression data, obtained from PBMC of 1,933 subjects of our cohort of patients with CAD^20^. While no transcripts were significantly associated with the 9p21 genotype at a genome-wide level^17^, gene set enrichment analyses of *circANRIL*-correlated genes (*n*=1,864; *p*≤0.001 (Ingenuity Pathway Analysis)) revealed induction of p53 signalling as the most significantly enriched pathway (Table 1) demonstrating that *circANRIL* also promoted nucleolar stress *in vivo*. Taken together, genome-wide expression and pathway analyses in large human CAD cohorts, pre-rRNA expression levels in human atherosclerotic plaques and functional studies in relevant human cells validate a circRNA effector mechanism, where *circANRIL* controls pre-rRNA maturation and nucleolar stress and thereby may act as protective factor against human atherosclerosis.

## Discussion

Our work provides a proof of concept for a long non-coding circRNA as a molecular regulator of rRNA maturation and of key cellular functions relevant to human disease. Results of this study extend our current knowledge about the molecular mechanisms of circRNAs, previously including the escape from translation^8^ and miRNA sponging^2^,^9^, to control of ribosomal RNA maturation through circRNA-protein interaction. We show that *circANRIL* binds to the C-terminal lysine-rich domain of PES1, thereby preventing pre-rRNA binding and exonuclease-mediated rRNA maturation^25^. Consequently, *circANRIL* impairs ribosome biogenesis, leading to activation of p53 and a subsequent increase in apoptosis and decrease in proliferative rate. Together, this pathway may promote atheroprotection by culling overproliferating cell types in atherosclerotic plaques.

PES1 is a nucleolar protein that is known to assemble in the PeBoW complex, which consists of PES1, BOP1 and WDR12. This complex is central for ribosome biogenesis and essential for life from yeast to mammals^26^,^36^. Additional support for an important role or the PeBoW complex in human atherosclerosis comes from genome-wide association studies of CAD, which independently identified WDR12 as an atherosclerosis modifier gene^13^,^36^,^37^. Human PES1 is a 588 amino-acid protein^38^, and previous work has shown that the central BRCT motif is essential for the incorporation of PES1 into the PeBoW complex and nucleolar localization^30^. In contrast, deletion of the C-terminal domain, containing NLS motifs, did not affect nucleolar localization but blocked rRNA maturation^29^. This was accompanied by changes in nucleolar morphology, inhibition of proliferation and induction of p53 in proliferating cells^29^. Results of the current work establish the C-terminal domain of PES1 as the binding site of both *circANRIL* and pre-rRNA. *circANRIL* binding prevented exonuclease-mediated processing of pre-rRNA, causing a dominant-negative phenotype as observed for the C-terminal deletion mutants of PES1 (Fig. 7g-j). It is remarkable that *circANRIL* can interfere with rRNA processing despite the relatively low abundance of detectable *circANRIL* RNA in the cell. On the basis of the published copy number of *CDR1as* in HEK-293 cells^2^ and our finding that *circANRIL* is 4–6 times higher expressed than *CDR1as* in these cells (Supplementary Fig. 2a), we estimate that the copies of *circANRIL* RNA per HEK cell will amount to 800–1,000. As a working model of *circANRIL* function, the stoichiometry of *circANRIL* copies relative to copies of PES1 protein might determine the efficacy of blocking PES1-dependent rRNA processing. Assuming a median abundance of 8,000 protein molecules per cell for an average protein in a typical tissue culture line^39^, a stoichiometry of *circANRIL* RNA:PES1 protein of 1:10 can be estimated. These data suggest that PES1 might be inactivated by *circANRIL* at a certain percentage even in native, non-overexpressing cells. One might speculate that thereby, *circANRIL* may impair PES1-mediated rRNA processing and ultimately impair protein translation rate and cell growth. We also base this hypothesis on a previously published study where a dominant-negative phenotype has been revealed for a C-terminal deletion mutant of PES1, such that mutant PES1 incorporated into the PeBoW complex inactivated the complex and blocked processing of the 32S pre-rRNA, leading to stabilization of p53 and to cell cycle arrest^29^. Notably, we also detected a mosaicism of *circANRIL* expression levels in primary SMC and macrophages in vascular tissues (Fig. 1), suggesting that *circANRIL*'s roles in curbing atherogenesis may have to be investigated on a single-cell level to fully appreciate *circANRIL* function.

Despite the fact that we overexpressed *circANRIL* only threefold in HEK-293 cells, it is also evident that the physiologically occurring differences in *circANRIL* expression observed in our human studies were smaller than in the overexpressing cell lines. Consequently, they are likely to be accompanied by smaller biological effects as well. Nevertheless, it is important to recognize that effects in overexpressing cell lines could be replicated in human primary cells with the same directions of effects (Fig. 8). Moreover, we identified the same molecular effector mechanisms of *circANRIL* in stably overexpressing cell lines and human cohorts, where high *circANRIL* led to the accumulation of pre-rRNA and p53 activation. Since atherosclerosis develops throughout life, it is plausible that subtle genotype-directed gene expression differences modulate the risk to develop symptomatic atherosclerotic cardiovascular disease at a higher age. Therefore, studies in overexpressing cell lines might be viewed as a time-lapse of the protective effects of *circANRIL* seen in individuals carrying the 9p21 atheroprotective genotype free of atherosclerosis (Figs 1 and 8).

Recently, substantial progress has been made in understanding the molecular mechanism of RNA circularization. It is thought that exonic circRNAs are generated by so called ‘back-splicing', enhanced by complementary base pairing of inverted repeats in the circRNA flanking introns^1^,^5^,^6^,^19^,^40^,^41^. Current work has also revealed a growing number of proteins affecting exon circularization^41^,^42^,^43^, and the proportion of circular versus linear transcripts may depend on the presence of specific intronic binding sites for these factors^44^. Even though the mechanism of *ANRIL* circularization has not been the scope of this work, it is likely that single-nucleotide polymorphisms contained in the 9p21 haplotype are responsible for differential *circANRIL* formation. Since low *linANRIL* and high *circANRIL* expression was associated with atheroprotection at 9p21, our results also underscore the importance of a balanced expression of linear and circular RNA transcripts. More generally, our results support a concept where circularization might be protective to escape linear RNA function, which has been associated with the onset and progression of diseases^16^,^17^,^45^.

Soon after the identification of the 9p21 locus by human genome-wide association studies^12^,^13^, a mouse model with a 70 kb deletion at chromosome 4 (Chr4^Δ70kb/Δ70kb^), representing the mouse orthologue of the human 9p21 risk haplotype, has been published^46^. While the deletion had no effect on atherosclerosis, *in vitro* studies revealed increased proliferation of isolated aortic SMC from Chr4^Δ70kb/Δ70kb^ animals^46^. The direction of effects was comparable to our results of the knockdown of *circANRIL*, which increased proliferation as well. It is, however, unlikely that the effects observed in the Chr4^Δ70kb/Δ70kb^ mouse model are related to *ANRIL*, because phylogenetic analyses revealed that *ANRIL* is absent in mice and fully developed only in certain primates, including humans^18^. In line with this, we detected no *ANRIL* expression in mouse tissue, and therefore, mouse models are not informative for the study of *ANRIL.* Instead, results of our study support a recent gain of function of primate-specific non-coding circRNA in controlling evolutionary conserved protein complexes. Several RNAs without evident protein-coding function were shown to co-localize with ribosomes^47^, suggesting that regulation of ribosome biogenesis by lncRNAs might be a broader phenomenon in humans than previously anticipated.

But how may *circANRIL*-mediated inhibition of rRNA processing be linked to atheroprotection? A major finding of the current study was that *circANRIL* induced ‘nucleolar stress' (Figs 6, 7) with subsequent stabilization of p53. Oncologists are now considering nucleolar stress strategically as a potential anticancer therapy^32^,^48^. In a similar fashion as cancer, atherosclerosis may be viewed as a proliferative disease. However, the role of proliferation and apoptosis in atherosclerosis is multi-facetted and inhibition of cellular proliferation and induction of apoptosis is not necessarily atheroprotective under all circumstances. For instance, while apoptosis in endothelial cells (EC) is proatherogenic, apoptosis in macrophages might attenuate early progression but can also induce secondary inflammation and plaque rupture^49^. The net effect of these individual mechanisms is difficult to determine experimentally. Nevertheless, one of our key observations was that p53 was induced on *circANRIL* overexpression, and mouse models have demonstrated that p53 is atheroprotective^50^,^51^, providing further evidence for an atheroprotective net effect of *circANRIL*.

Taken together, our data suggest *circANRIL* as a potential therapeutic target for the treatment of atherosclerosis, since it might promote antiatherogenic cell functions and is particularly stable against degradation. High stability seems to be a common feature of circRNAs^1^,^19^, which might therefore serve as attractive novel therapeutic targets for human diseases in more general terms.

## Methods

### Study cohorts

Association analysis of gene expression was performed in samples of the LIFE Heart Study (*n*=2,280), a cross-sectional cohort study of patients undergoing coronary angiography for suspected CAD^20^. The study has been approved by the Ethics Committee of the Medical Faculty of the University Leipzig (Reg. No 276-2005). Human endarterectomy specimens (*n*=218) were collected in a cohort of patients undergoing vascular surgery, and the utilization of human vascular tissues was approved by the Ethics Committee of the Medical Faculty Carl Gustav Carus of the Technical University Dresden (EK316122008)^17^,^52^. Human primary cells (EC, SMC and adventitial fibroblasts (FB)) from non-diabetic, non-smoking patients and liver samples were obtained and experimental procedures were performed within the framework of the non-profit foundation Human Tissue and Cell Research^53^. Written informed consent was obtained from all subjects.

### DNA and RNA isolation and 9p21 genotyping

Isolation of DNA and RNA from PBMC, human primary tissues and cells was performed using TRIzol reagent (Thermo Fisher Scientific)^16^,^17^. RNA from nuclear and cytoplasmic fractions and whole-cell lysates for RNA distribution studies were prepared using the PARIS Kit (Ambion) according to the manufacturer's instructions. Chr9p21 protective (A) and risk (G) haplotypes (Fig. 1d,e) were defined by single nucleotide polymorphisms rs10757274, rs2383206, rs2383207 and rs10757278 (refs 16, 52).

### Reverse transcription

RNA from human samples and cell lines was reverse transcribed using random hexamer primers (Roche). For validation of circular RNA overexpression, RNA was reverse transcribed with random hexamer primers (Roche Life Sciences), detecting linear and circular RNA transcripts and oligo(OdT) primers (Roche Life Sciences), for detecting linear transcripts containing poly-A tails. For miRNA quantification, RNA was reverse transcribed using the miScript II RT Kit (Qiagen).

### PCR and qPCR

The AmpliTaq DNA Polymerase (Life Technologies) was used for PCR and qPCR reactions. PCR reactions for amplification of *circANRIL* were performed in cDNA from SMC, FB, EC, MonoMac and HEK-293 cells, with outward-facing primers in *ANRIL* exon 6 (ref. 14). No PCR products were amplified in EC. PCR products were cloned using the TOPO TA Cloning Kit (Life Technologies) and sequenced with an automated DNA sequencer (Applied Biosystems). Quantification of gene expression was performed on a ViiA 7 Real-Time PCR System (Life Technologies) according to published protocols and normalized to plasmid standard curves^17^. PCR products were visualized using the Lonza FlashGel System (Biozym). Primers and probes for amplification of *linANRIL* (synonymous to *ANRIL ex18-19* in ref. 17), *U1*, beta actin (*BA*), *GAPDH*, *CDKN2A* (*p14*^*ARF*^*, p16*^*INK4a*^), *CDKN2B* (*p15*^*INK4b*^)^17^, *MALAT1* (ref. 54) and *CDR1as* (ref. 2) were used according to published protocols. Primers and probes for *circANRIL* (*ex7-5*), *lin+circANRIL* (*ex6-7*), *circHPRT1* (*ex6-2*), *lin+circHPRT1* (*ex2-3*), *PES1*, sex-determining region Y-box 2 (*SOX2*) and v-maf avian musculoaponeurotic fibrosarcoma oncogene homologue (*MAF*)^55^ are given in the Supplementary Table 9. Expression of 47S, 36S and 32S rRNA and 7SL RNA control was determined according to ref. 31 using the KAPA SYBR FAST reagent (PeqLab). Quantification of miRNAs Hs_miR-3184-3p_1 (MS00041944, Qiagen), Hs_miR-4659b-3p_1 (MS00040390, Qiagen) and Hs_miR-5571-5p_1 (MS00038360, Qiagen) was performed with the miScript SYBR Green PCR Kit (Qiagen) and normalized to Hs_miR-27b_2 (MS00031668, Qiagen).

### Genome-wide expression profiling

Illumina HumanHT-12 v4 BeadChips arrays were used for expression profiling in human PBMC (*n*=2,280) of the LIFE Heart Study and in cell lines with stable *circANRIL* overexpression (*n*=3) and vector control (*n*=3). For data from cell lines, bead-level data preprocessing was done in Illumina GenomeStudio followed by quantile normalization and background reduction according to standard procedures in the software. Preprocessing of expression data in PBMC of the LIFE Heart Study comprised selection of expressed features, outlier detection, normalization and batch correction^17^. Gene expression data have been deposited at Gene Expression Omnibus (GSE65392).

### Expression vectors

*CircANRIL*, *circANRIL*-BoxB, BoxB and *circHPRT1* sequences were synthesized (Eurofins Genomics) with adjacent 5′- and 3′-140 bp intronic sequences (Fig. 2a and Supplementary Table 1) and cloned in the bicistronic pBI-CMV2 (Clontech) vector allowing parallel expression of a green fluorescent protein and RNA transcripts. Stable cell lines were generated through co-transfection with a neomycin-encoding vector^17^. PES1 wild type (PES1-WT) and truncated PES1 (PES1Δ1–54 and PES1Δ446–588, respectively; Fig. 6f and Supplementary Table 8) were cloned in the pmCherry-N1 vector (Clontech) encoding a neomycin resistance and allowing overexpression of PES1 fused to a red fluorescent protein (RFP).

### Cell culture and functional studies

HEK-293 (DMSZ, ACC305) were cultured in DMEM (Life Technologies) containing 10% fetal calf serum (FCS, Biochrom) and 1% penicillin/streptomycin (P/S; Life Technologies). MonoMac cells (DSMZ, ACC124) were cultured in RPMI 1640 (Biochrom) containing 10% FCS, 1% P/S, 1% MEM (Life Technologies) and 1% OPI (Sigma). Primary arterial SMC (*n*=5) and FB (*n*=5) were cultured in Smooth Muscle Cell Growth Medium 2 (PromoCell) and Fibroblast Growth Medium (PromoCell), respectively. iPSC were cultured using using mTeSR 1 Medium (STEMCELL Technologies)^56^ and differentiated into macrophages^55^. For generation of stably overexpressing cells, HEK-293 were transfected with *ANRIL*-pBI-CMV2 vectors (Clontech) and neomycin-encoding vector, cells were selected with geneticindisulfate (G418, Roth)^17^ and overexpression was validated using specific qPCRs. At least three cell lines were generated per isoform and vector control, respectively. Knockout of the 9p21 locus in HEK-293 cells was accomplished using CRISPR/Cas9 technology^56^ with guide RNAs (Fig. 3a and Supplementary Table 10) and controlled using PCR and sequencing (Fig. 3a,b; Supplementary Tables 2 and 10). Transient transfection of HEK-293 9p21 mutant, primary SMC and iPSC-derived macrophages was performed using Lipofectamine 2000 (Life Technologies) or Nucleofector technology (Lonza) according to the manufacturer's instructions. Cellular proliferation was determined using CellTiter-Glo Luminescent Cell Viability Assay (Promega). Apoptosis was induced by staurosporine (0.25 and 0.5 μM; Calbiochem) and quantified by a caspase-3 activity assay (Caspase-Glo 3/7 Assay; Promega). Measurements were performed on the SpectraMax Paradigm Multi-Mode Microplate Reader (Molecular Devices). Transfection of siRNA was performed using Lipofectamine 2000 (Life Technologies) or Nucleofector technology (Lonza) according to the manufacturer's instructions. The following siRNAs were used for downregulation of *ANRIL* isoforms: *non-circANRIL*: n272158, exon 1 (Life Technologies); *circANRIL*: 2046125, exon 7 (Qiagen); *GAPDH* (4390849; Life Technologies); and SCR control (Silencer Select Negative Control No. 1 siRNA, 4390843; Life Technologies). siRNA-mediated downregulation of *ANRIL* or overexpression of *ANRIL* and PES1 isoforms was validated by isoform-specific qPCRs and western blotting, respectively. All experiments were performed in quadruplicates using 2–4 biological replicates. To assess RNA stability, cells were incubated with actinomycin D (50 ng ml^−1^; Sigma) for up to 72 h. For pre-mRNA splicing inhibition, cells were incubated with isoginkgetin (Calbiochem) for 24 h.

### Antibodies

Antibodies for western blotting, immunofluorescent staining and RIP, and secondary antibodies are given in Supplementary Table 11.

### Western and northern blotting

Nuclear and cytoplasmic fractions and whole-cell lysates for western blotting were prepared using the PARIS Kit (Ambion) and run on NuPAGE Novex 4–12% Bis-Tris Protein Gels (Life Technologies). Primary and secondary peroxidase-labelled antibodies are given in Supplementary Table 11, AceGlow-Solution (PeqLab) was used for visualization of the chemiluminescent light signal of protein bands (white on black background) using the Fusion-FX7 Advance system (PeqLab). For northern blot analysis, 2 μg of total RNA isolated from HEK-293 cell lines with stable *circANRIL* or vector overexpression were separated on an 1% agarose gel containing formaldehyde and blotted onto a Hybond-N+ membrane (GE Healthcare). rRNA molecules were detected using the 3′-BITEG-labelled probes ITS1 and ITS2 (ref. 36) and streptavidin-POD (Roche). Western and northern blots were repeated three times and representative experiments are shown in the manuscript.

### *In situ* hybridization and immunohistochemical stainings

*In situ* RNA hybridization was conducted in formaldehyde-fixed endarterectomy tissues with digoxygenin (DIG)-labelled RNA probes (Roche Life Sciences) against *circANRIL* (exons 7–5–6; 381 bp) and sense negative control according to ref. 57. Anti-DIG-AP (Roche Life Sciences) was used as secondary antibody and staining was developed with NBT/BCIP solution (Roche Life Sciences). For dot blot analysis (Supplementary Fig. 12), RNA was spotted onto a Hybond-N+ membrane (GE Healthcare) and detected using a DIG-labelled probe against *circANRIL* and the anti-DIG-HRP antibody (PerkinElmer). Immunohistochemical staining of CD68 and SMA was performed using the ImmPRESS HRP Anti-Rabbit and Anti-Mouse Ig Polymere Detection System (Vector Laboratories) and 3,3′-diaminobenzidine^52^. *In situ* hybridization and immunohistochemical stainings were performed three times and representative images are shown in the manuscript.

### Immunofluorescent stainings

Cells were fixed with 1% formaldehyde and permeabilized with 0.3% Triton X-100. After blocking with 5% goat normal serum (Jackson ImmunoResearch Laboratories), primary antibodies were applied overnight at 4 °C at a dilution 1:100. Secondary antibodies were applied at a dilution 1:500 for 1 h at room temperature. Nuclei were stained with Hoechst 33342 (Sigma Aldrich) and samples were mounted with Fluorescence Mounting Medium (Dako). Pictures were taken with an Olympus Fluorescence Microscope OlympusBX40 and an Olympus XM10 camera. Immunoflurescent stainings were performed three times and representative images are shown in the manuscript. Nucleoli were counted per cell and nucleolar size was determined in px^2^ with the LAS V4.2 software (Leica).

### λN-Peptide-mediated pull-down of *circANRIL-*bound proteins

Pull-down of *circANRIL*-BoxB (Supplementary Fig. 3; ref. 23) was performed with the N-terminal domain (amino acids 1–22) of the λN-peptide coupled to *d*-desthiobiotin (Intavis Peptide Services) according to ref. 58. As controls, cellular lysates from cell lines with stable overexpression of *circANRIL* were used. Streptavidin beads (Dynabeads MyOne, Life Technologies) were prewashed according to the manufacturer's instructions and blocked with 1 mg tRNA per ml and 1 mg BSA per ml in LN-buffer (20 mM Tris-HCl (pH 7.5), 0.1% NP40, 150 mM NaCl, 1 mM MgCl_2_, 0.2 mM EDTA and 1 mM dithiothreitol). Cellular lysates (1 mg total protein) were incubated with 100 μl streptavidin beads overnight at 4 °C in 1,000 μl LN-buffer, 100 μg tRNA per ml, 1 U RNasin per ml and 10% glycerine. After washing with 5 × with LN-buffer, 3 × with LN-buffer, 500 mM NaCl and 2 × with LN-buffer containing 250 mM LiCl instead of NaCl, *circANRIL*-BoxB-bound proteins were eluted with eluted with 100 μl Urea buffer (2 M urea and 50 mM Tris (pH 7.5)) supplemented with 1 mM dithiothreitol and 5 μg μl^−1^ trypsin and LysC. After alkylation with 5 mM iodoacetic acid (IAA), proteins were proteolytic digested with trypsin and LysC for 24 h. Peptides were acidified, loaded on SDB-RPS material and eluted and dried^59^. Peptides were resuspended in buffer A* (2% acetonitrile (ACN) and 0.1% trifluoroacetic acid (TFA)) and were briefly sonicated (Branson Ultrasonics, Ultrasonic Cleaner Model 2510) before mass spectrometric analyses. *CircANRIL*-BoxB RNA was eluted with 1% SDS, 20 mM Tris-HCl (pH 7.0), 0.1 mM EDTA at 99 °C for 15 min, extracted with TRIzol (Life Technologies)/CHCl_3_ and precipitated with isopropanol. RNA was reverse transcribed with random hexamer primers and analysed by qPCR.

### RNA immunoprecipitation

RIP was performed using antibodies given in Supplementary Table 11 (ref. 17). RFP-Trap_M (rtm-20, Chromotek) was used for immunoprecipitation of PES1-mCherry fusion proteins using incubation and washing buffers as for the λN-peptide-mediated pull-down. RNA was extracted with TRIzol (Life Technologies)/CHCl_3_ and precipitated with isopropanol. Retrieved RNA was reverse transcribed using random hexamer primers and analysed by qPCR with primers specific for *circANRIL* (*ex7-5*) or controls (*CDR1as*, *circHPRT1*).

### PulseSILAC

*CircANRIL*-overexpressing and control cells were attenuated in ‘light' medium (Silantes) for three passages. After seeding and adhesion for 1 h, cells were switched to ‘medium' (SILAC-Lys4-Arg6-Kit, Silantes) and ‘heavy' medium (SILAC-Lys8-Arg10-Kit, Silantes) and incubated for 24 h (Fig. 4h). After washing with PBS, cell were lysed in SDC buffer (1% SDC, 10 mM TCEP, 40 mM CAA and 100 mM Tris (pH 8.5)), boiled for 10 min at 95 °C, sonicated and diluted 1:1 with ddH_2_O before proteolytic digestion. Peptide purification was done^59^ before mass spectrometric analyses.

### Mass spectrometric data acquisition and data analyses

A unit of 2 μg of peptides from circANRIL-BoxB and circANRIL control pull-down experiments (*n*=4/4) were loaded for 30 min gradients separated on a 20 cm column with 75 μm inner diameter in-house packed with 1.9 μm C18 beads (Dr Maisch GmbH). A unit of 2 μg of peptides from pulseSILAC experiments were loaded for 100 min gradients separated on a 40 cm column with 75 μm inner diameter in-house packed with 1.9 μm C18 beads (Dr Maisch GmbH). Reverse phase chromatography was performed at 60 °C with an EASY-nLC 1,000 ultra-high pressure system (Thermo Fisher Scientific) coupled to the Q Exactive Plus mass spectrometer (for RNA pull-downs) or to the Q Exactive HF mass spectrometer (for pulseSILAC experiments) (Thermo Fisher Scientific) via a nanoelectrospray source (Thermo Fisher Scientific). Peptides were loaded in buffer A (0.1% (v/v) formic acid) and eluted with a nonlinear gradient. Operational parameters were real-time monitored by the SprayQC software^60^. Raw files were analysed by MaxQuant software (version 1.5.0.0 (RNA pull-downs) or version 1.5.0.38 (pulseSILAC experiments))^61^, and peak lists were searched against the *Homo sapiens* Uniprot FASTA database (Version 2014/4) and a common contaminants database (247 entries) by the Andromeda search engine^62^. Label-free quantification was done using the MaxLFQ algorithm^63^ integrated into MaxQuant. Data analysis was performed with the Perseus software in the MaxQuant computational platform and in the R statistical computing environment. Data were imputed by creating a Gaussian distribution of random numbers with a s.d. of 30% in comparison to the s.d. of measured values, and one s.d. downshift of the mean to simulate the distribution of low signal values.

### miRNA-binding and RNA–protein interaction prediction

Predictions of miRNA-binding sites were performed using miRanda^64^ and TargetSpy^65^ algorithms. miRNA sequences were downloaded from miRBase (Release 21 (ref. 66)). The catRAPID^67^ and the RNA-Protein Interaction Prediction (RPISeq) software^67^,^68^ were used for predictions of protein–RNA binding. Secondary RNA structure prediction was performed using the Vienna RNA package^69^.

### Pathway and upstream regulator analyses

Genome-wide expression profiling revealed 1,864 *circANRIL*-correlated transcripts in human PBMC (*n*=2,280; *P*<0.001) and 18,09 transcripts with >2- or <0.5-fold expression changes in *circANRIL*-overexpressing compared with vector control cells. Using pulseSILAC, 197 proteins with *de novo* synthesis ±10% in *circANRIL*-overexpressing compared with control cell lines were identified. Pathway and upstream regulator analyses were performed using the Ingenuity Pathway Analysis (www.ingenuity.com) according to standard procedures of the software. Levels of significance were determined using Fisher's exact tests implemented in the software. Bonferroni corrections for the number of tests were applied, and *P* values with genome-wide significance are given in Supplementary Tables 5 and 6.

### Statistics

Normality of distribution was tested using the Kolmogorov–Smirnov test implemented in the PRISM statistical software (GraphPad). Comparison of normally distributed multiple groups was done using analysis of variance and Tukey was performed as post-test. Comparison of two normally distributed groups was done using a *t*-test. Comparison of two non-normally distributed groups was done using the Mann–Whitney *U*-test. Mendelian randomization analysis and associations of haplotypes with gene expression and of *circANRIL* with atherosclerosis were calculated using the R software for statistical computing^16^,^17^,^70^.

### Data availability

Gene expression data have been deposited at Gene Expression Omnibus (http://www.ncbi.nlm.nih.gov/geo/) under the GEO Accession number GSE65392. The mass spectrometry proteomics data have been deposited to the ProteomeXchange Consortium via the PRIDE partner repository (https://www.ebi.ac.uk/pride/archive/) with the data set identifier PXD001769. Original uncropped scans of DNA agarose gels and of western blots have been assembled in Supplementary Figs 17 and 18, respectively. All other data are available on request.

## Additional information

**How to cite this article:** Holdt, L. M. *et al.* Circular non-coding RNA *ANRIL* modulates ribosomal RNA maturation and atherosclerosis in humans. *Nat. Commun.* 7:12429 doi: 10.1038/ncomms12429 (2016).

## Supplementary Material

Supplementary Information Supplementary Figures 1- 18 and Supplementary Tables 1-11

## Figures and Tables

**Table 1:** Upstream regulator analysis of *circANRIL*-correlated transcripts in PBMC of the LIFE Heart Study (*n*=2,280).

Transcription regulator	*P-*value
TP53	7.8 × 10^−09^
E2F1	1.8 × 10^−07^
STAT5A	2.9 × 10^−07^
IRF8	5.2 × 10^−07^
STAT1	5.8 × 10^−06^
SMARCA4	6.5 × 10^−06^
ELF4	1.5 × 10^−05^

Transcripts correlated with *circANRIL* expression (*P*<0.001; *n*=1,864, Ingenuity Pathway Analysis) were included in the analysis.
