# Supplementary material for: Circular non-coding RNA ANRIL modulates ribosomal RNA maturation and atherosclerosis in humans
Source: Nat Commun. 2016 Aug 19;7:12429. doi: 10.1038/ncomms12429 (PMC4992165; doi:10.1038/ncomms12429)
Supplement: Supplementary Information — Supplementary Figures 1- 18 and Supplementary Tables 1-11 [file ncomms12429-s1.pdf]

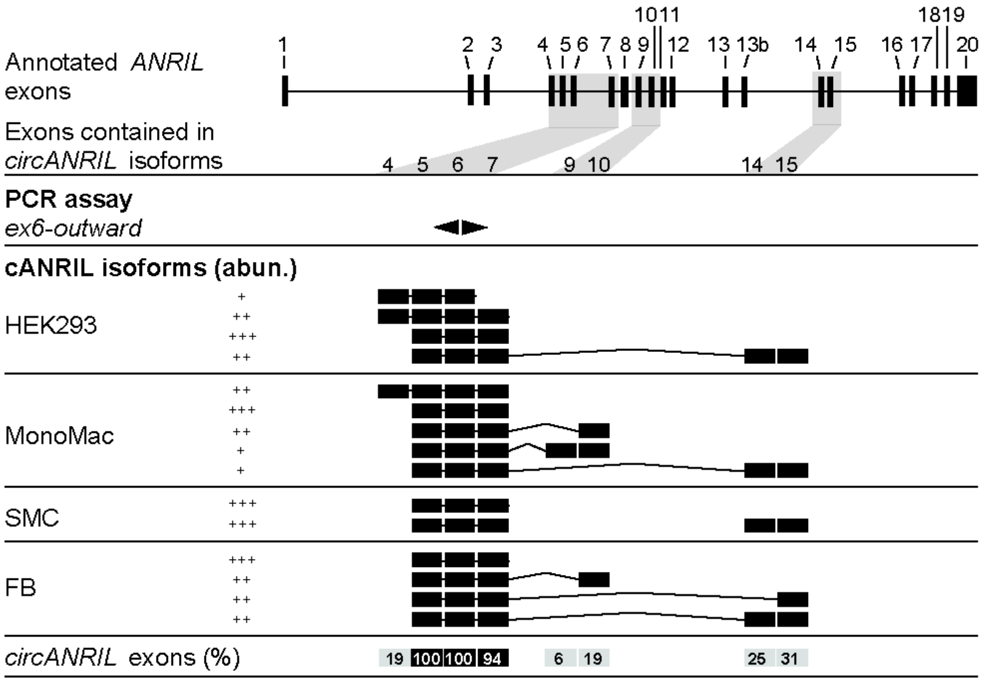

Supplementary Figure 1. Investigation of *circANRIL* transcript structure in human cell lines and primary cells. Annotated *ANRIL* exons and location of outward-facing PCR primers in exon 6. *CircANRIL* isoforms identified in human embryonic kidney cell line (HEK-293), human monocytic cell line (MonoMac), primary smooth muscle cells (SMC), and adventitial fibroblasts (FB). No exon scrambling except for back-splicing events was detected in *circANRIL* isoforms. abun. – abundancy; ++ - at least two sequences, +++ - most abundant isoforms. *CircANRIL* consisting of exons 5, 6, 7, where exon 7 is back-spliced to exon 5, was the most abundant isoform detected in all investigated cells and was selected for functional studies.

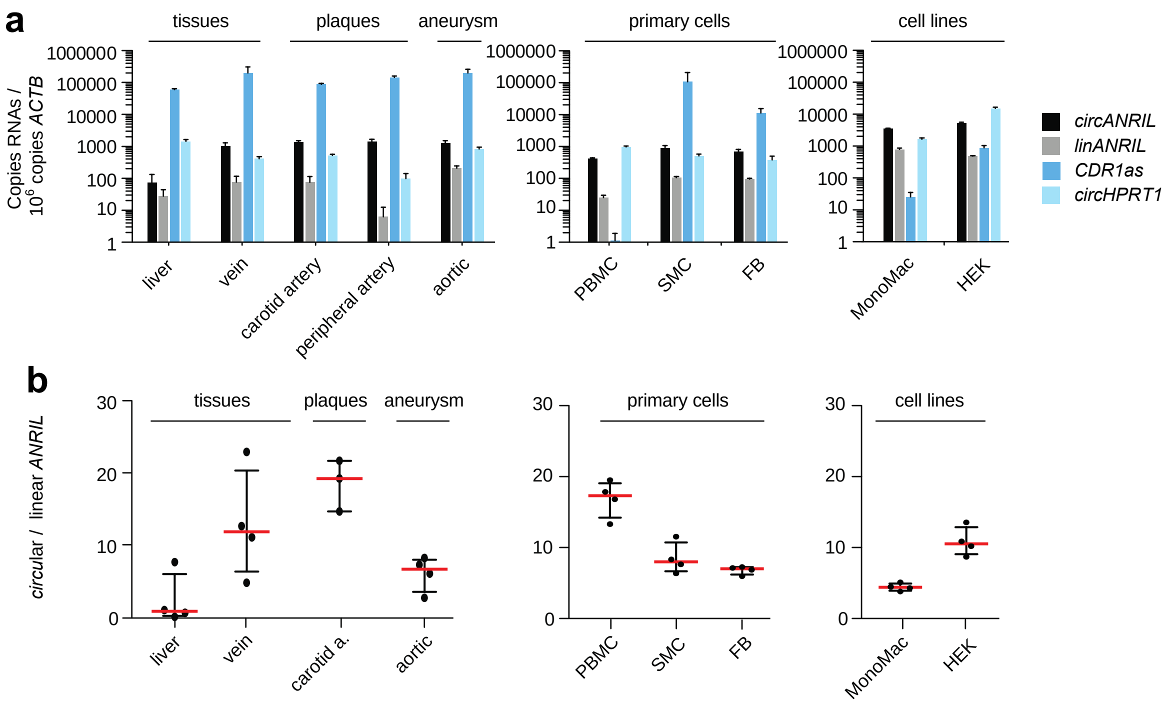

Supplementary Figure 2. RNA abundance of different circular RNAs in tissues and cells. **(a)** Copies of *circANRIL*, circular *CDR1as*, *circHPRT1*, and of linear *ANRIL* (*linANRIL*) RNA in human tissues, primary vascular cells, and cell lines normalized to 10^6 copies of housekeeping gene *ACTB*. Slight differences of *circANRIL* expression levels compared to Fig. 1b are explained by the use of a new batch of reverse-transcribed cDNAs. Peripheral blood mononuclear cells (PBMC), smooth muscle cells (SMC), adventitial fibroblasts (FB), human monocytic cell line MonoMac, human embryonic kidney cell line HEK-293. **(b)** Ratio of circular/linear *ANRIL* in tissues (left panel), primary cells (middle panel), and cell lines (right panel). Ratios were not calculated for plaques of peripheral arteries, for ECs, and for the THP1 cell line due to absence or very low expression of *linANRIL* in these samples. Red lines indicate median of ratios. Interquartile ranges are shown.

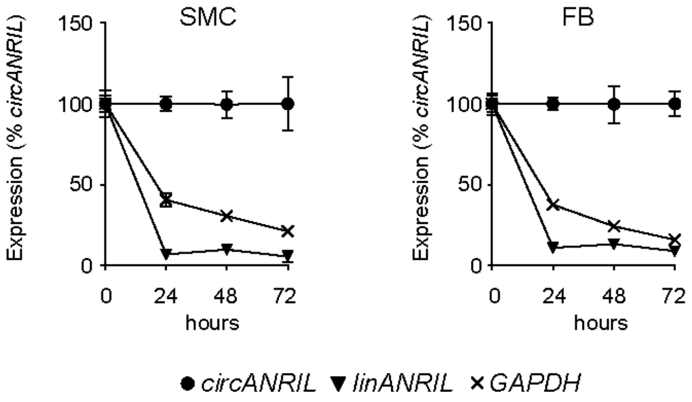

Supplementary Figure 3. *CircANRIL* is more stable than *linANRIL* in human primary cells. Half-lives of *circANRIL*, *linANRIL*, and of house-keeping *GAPDH* mRNA were determined in smooth muscle cells (SMC) and adventitial fibroblasts (FB) by qPCR analysis following inhibition of transcription with 50ng/ml actinomycin D. Analyses were performed in triplicates in RNA of 3 biological replicates. Data are given as mean ± s.e.m.

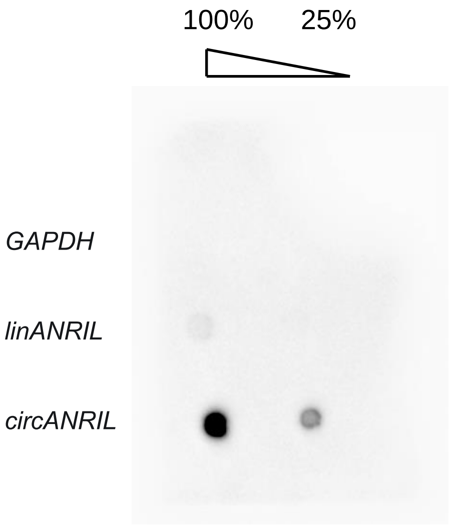

Supplementary Figure 4. Dot blot analysis of DIG-labeled probe that was subsequently used for *in situ* hybridization in Fig. 1c. RNAs for *GAPDH*, *linANRIL*, and *circANRIL* were spotted on a nylon membrane (*GAPDH*, *circANRIL* (line 1 5 fmol each) *linANRIL* (line 1 0,5 fmol resembling observed expression differences in Supplementary Fig. 2b)) and hybridized with the DIG-labeled probe.

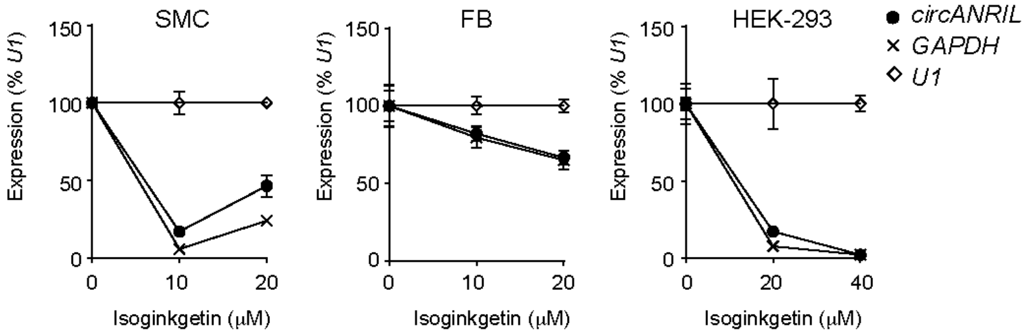

Supplementary Figure 5. Spliceosome-dependent generation of *circANRIL*. Reduced generation of *circANRIL* after incubation of smooth muscle cells (SMC), adventitial fibroblasts (FB) and HEK-293 cells with isoginkgetin, a general splicing inhibitor. Data were derived from quadruplicate measurements per condition. *U1* is not spliced and was used as reference RNA. *GAPDH* mRNA generation is known to be spliceosome-dependent.

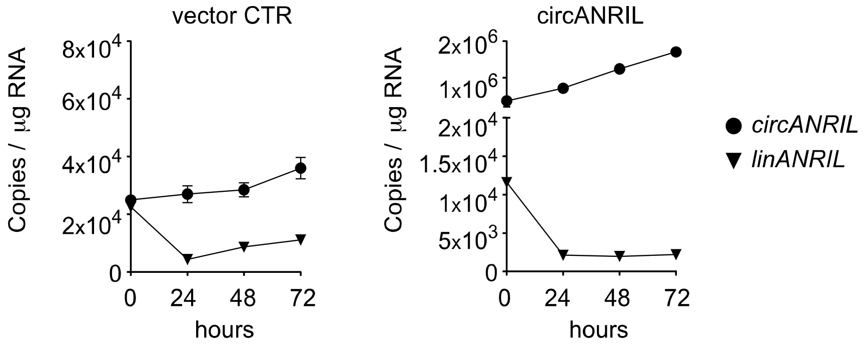

Supplementary Figure 6. *CircANRIL* is more stable than *linANRIL* in HEK-293 cells. Half-lives of *circANRIL* and of *linANRIL* were determined by qPCR analysis after inhibition of transcription with 50ng/ml actinomycin D in HEK-293 (left panel) or in HEK-293 cells overexpressing *circular* or *linear ANRIL*, respectively (right panel). Analyses were performed in triplicates in RNA of 3 biological replicates. Data are given as mean ± s.e.m.

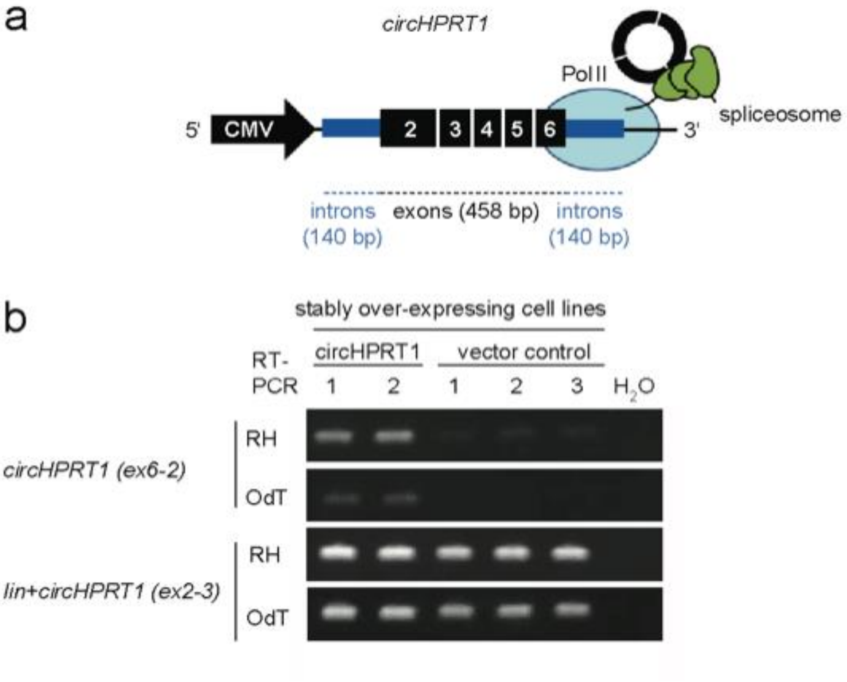

Supplementary Figure 7. Overexpression of *circHPRT1*. **(a)** Schematic of vector construct for expression of circular *HPRT1*. **(b)** Validation of *circHPRT1* overexpression by qPCR from random hexamer-primed (RH) or from oligo(d)T-primed (OdT) cDNA preparations. Results of biological replicates are shown on the agarose gel. Primer pair ex6-2 amplifies *HPRT1* RNA only after RNA circularization by back-splicing, primer pair ex2-3 recognizes both linear and circular *HPRT1* RNA.

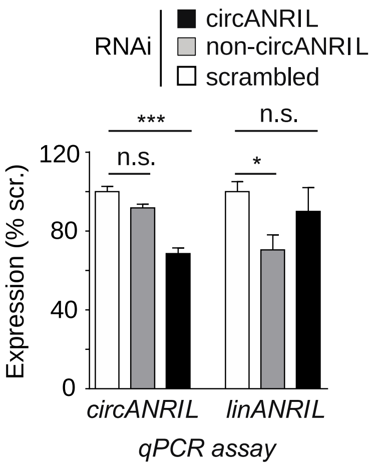

Supplementary Figure 8. Validation of linear or circular *ANRIL* siRNA-mediated down-regulation related to Fig. 2. qPCR Analysis was performed in quadruplicate measurements per condition. * $p < 0.05$; *** $p < 0.001$. Data are given mean ± s.e.m.

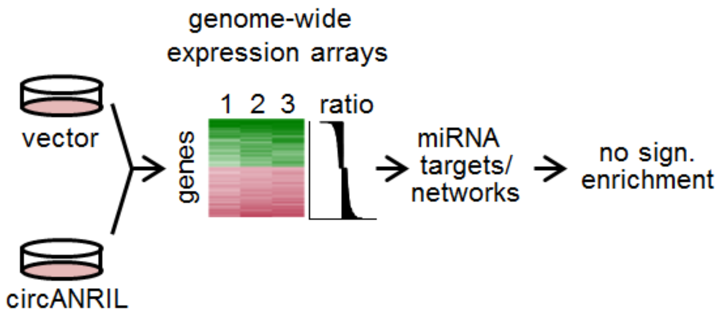

Supplementary Figure 9. Schematic of genome-wide expression analysis in *circANRIL* overexpressing (n=3) and vector control cells (n=3). Illumina HumanHT-12 v4 BeadChips were used for expression analyses. No significant regulation of miRNA-target gene networks was observed using the Ingenuity algorithm (www.ingenuity.com). Gene expression data have been deposited at Gene Expression Omnibus (GSE65392).

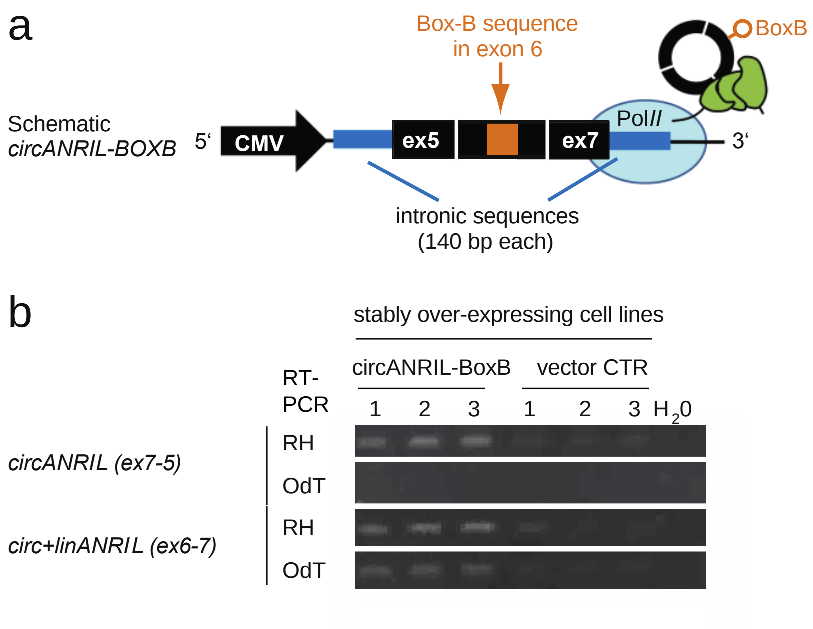

Supplementary Figure 10. Validation of *circANRIL* RNA containing BoxB RNA hairpin sequences. **(a)** Schematic of vector construct of *circANRIL* containing BoxB (*circANRIL-BoxB*). (**b**) Validation of *circANRIL-BoxB* overexpression by qPCR of random hexamer-primed (RH) or of oligo(d)T-primed (OdT) cDNA (3 biological replicates). Primer pair ex7-5 results in a PCR product only after RNA circularization by back-splicing, primer pair ex6-7 recognizes both linear and circular *ANRIL* RNA.

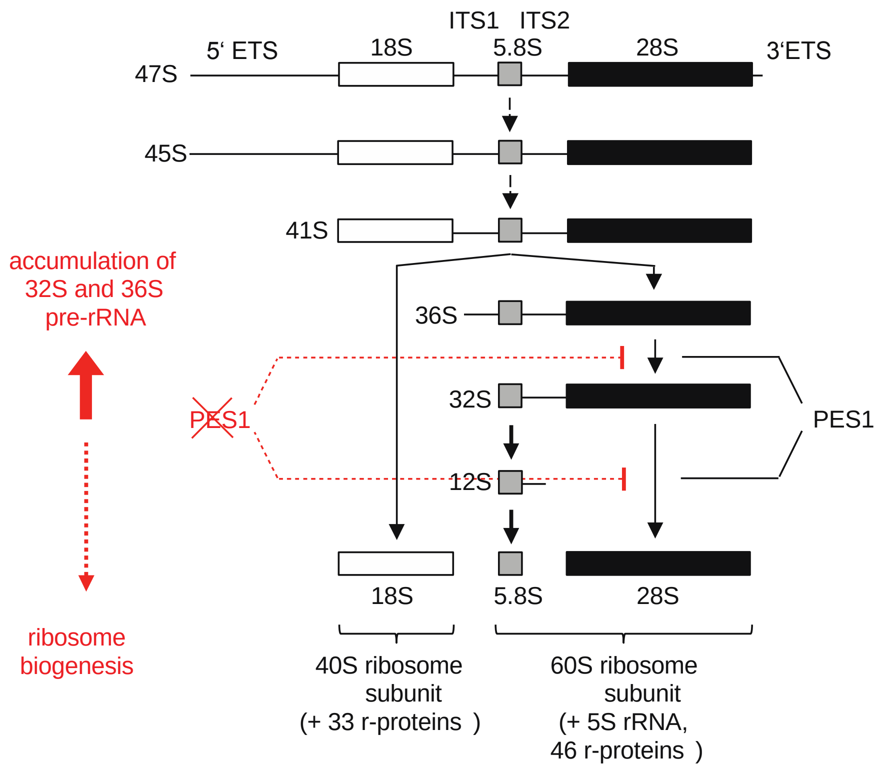

Supplementary Figure 11. Schematic of ribosomal RNA (rRNA) maturation and PES1 function. PES1 is required for 36S and 32S pre-rRNA processing to mature 5.8S and 28S rRNA. Impaired PES1 functions leads to accumulation of pre-rRNAs and reduced 60S ribosome biogenesis. ETS- external transcribed spacer; ITS- internal transcribed spacer.

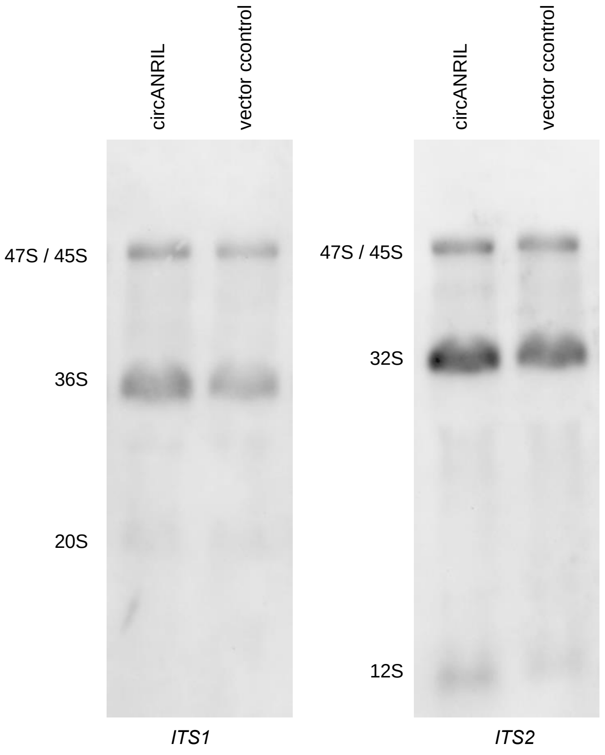

Supplementary Figure 12. Northern blotting in *circANRIL* overexpressing and vector control HEK-293 cell lines. *ITS1* and *ITS2* were used to detect pre-rRNA processing and pre-rRNA abundance in controls of following *circANRIL* RNA overexpression in HEK-293 cells.

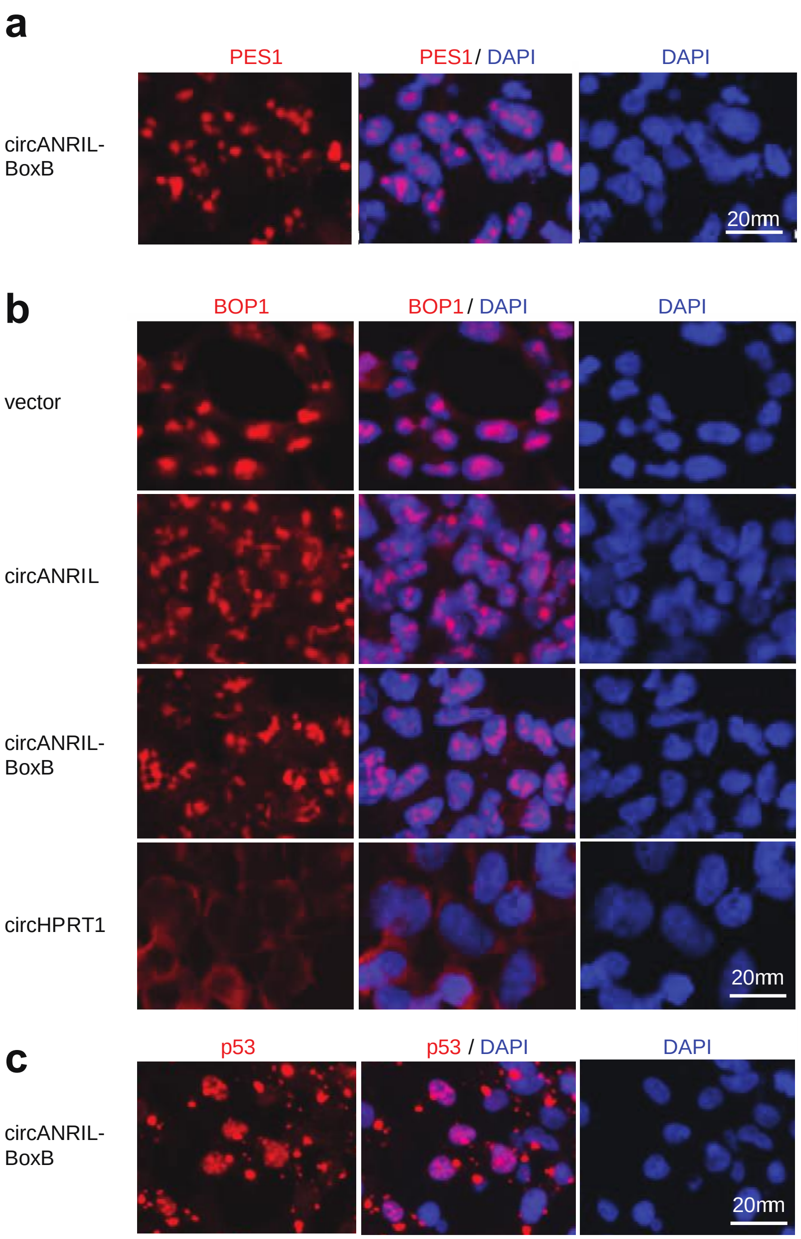

Supplementary Figure 13. Immunofluorescent stainings of PES1, BOP1, and p53 in *circANRIL* overexpressing and in control HEK-293 cells. (**a**) PES1 immunofluorescent staining in *circANRIL*-BoxB overexpressing cells corresponding to Fig. 6c. (**b**) BOP1 immunofluorescent staining in *circANRIL* overexpressing HEK-293 cells revealed altered nucleolar morphology. *circHPRT1* overexpression does not trigger nucleolar alterations. (**c**) p53 immunofluorescent staining in *circANRIL*-BoxB overexpressing HEK-293 cells corresponding to Fig. 6f.

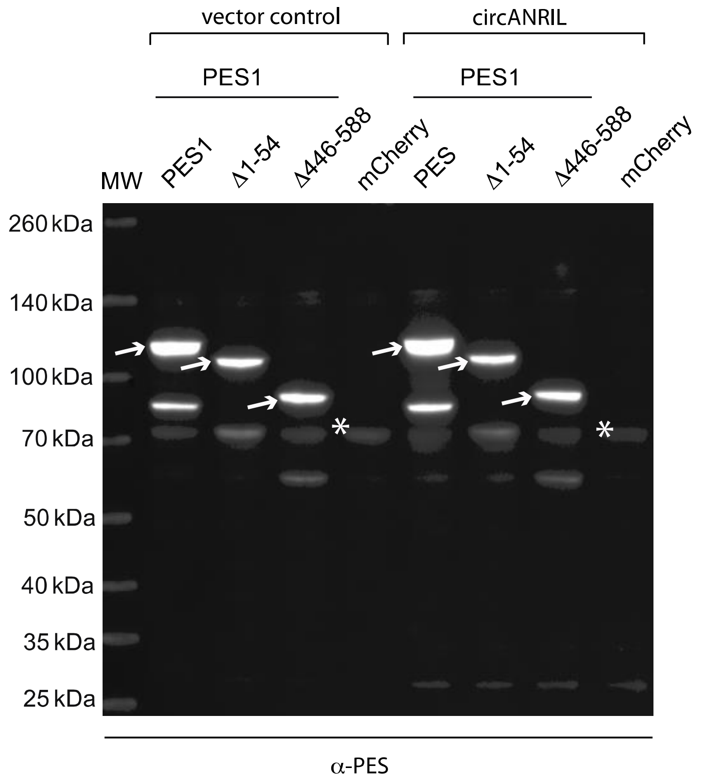

Supplementary Figure 14. Western Blot using PES1-specific antibody of protein extracts used for immunoprecipitation (IP) in Fig. 7e. The following constructs were expressed in HEK-293 cells: full length PES1 (PES1), the N-terminal deletion mutant lacking amino acids 1-54 (Δ1-54), the C-terminal deletion mutant lacking amino acids 446-588 (Δ446-588) or empty vector expressing mCherry alone. An asterisk indicates endogenous untagged PES1 protein.

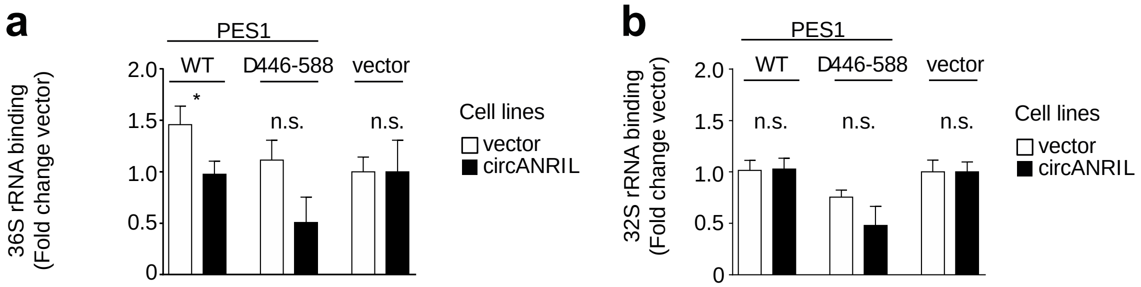

Supplementary Figure 15. Immunoprecipitation (IP) of PES1 isoforms and quantification of associated pre-rRNA by qPCR (**a**) 36S pre-rRNA binding to full lenght PES1 (PES1-WT) is significantly reduced when *circANRIL* is overexpressed. (**b**) 32S pre-rRNA binding to PES1 is not reduced by *circANRIL* overexpression. IP was performed in a pool of 3 biological replicates, qPCR measurements were performed in quadruplicates.

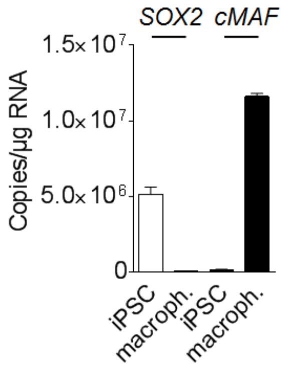

Supplementary Figure 16. Quantification of iPSC marker *SOX2* and macrophage differentiation marker *cMAF* in iPSC and iPSC-derived macrophages (macroph.) related to Fig. 8. Analysis was performed in quadruplicate measurements per condition. Data are given mean ± s.e.m.

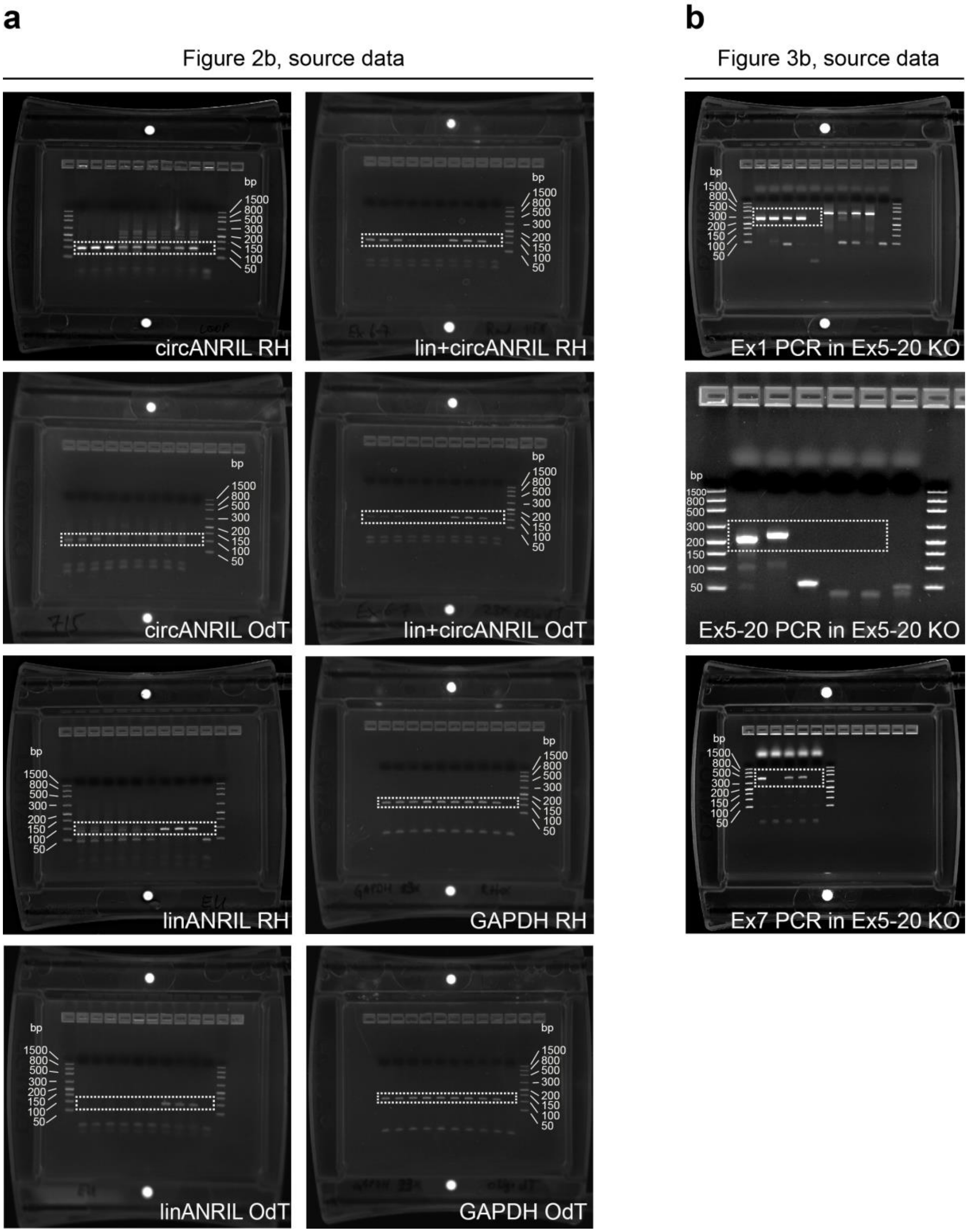

Supplementary Figure 17. Uncropped scans of DNA agarose gels used in Figures 2b and 3b. Regions that were selected for assembling the figures are indicated with dotted squares. Molecular size marker in base pairs (bp).

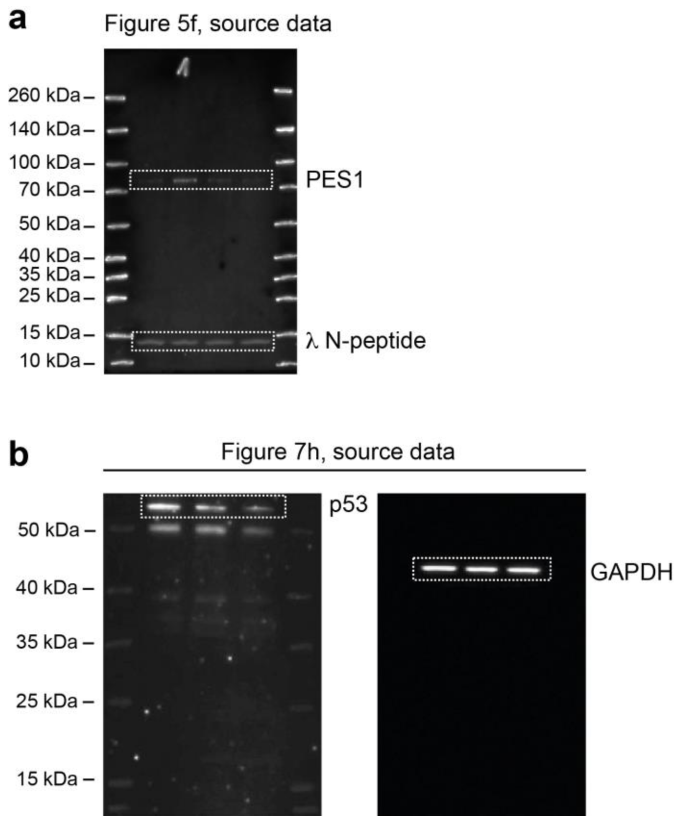

Supplementary Figure 18. Uncropped scans of Western blots used in Figures 5f and 7h. Regions that were selected for assembling the figures are indicated with dotted squares. The GAPDH blot was stripped and re-probed. Molecular weight marker in kilodaltons (kDa).

SUPPLEMENTARY TABLES

Supplementary Table 1. Plasmid sequences for circular RNA and BoxB overexpression.

>circANRIL(ex5-6-7), 689 bp without restriction sites, 409 bp exonic sequence

GAATTCGCGGCCGC*CAATGTATTTCTCTGCTTCATACACTATCCAAGGTGATAAAAGTGTTTATGAAGAG*
GGATTAGGAAATATCTGCTGTGTTTAGGTCATAGTCCAACCAAAGGATTAATTAACATTTGCCTCTTTTT
*TCTCCTACATCCAG*TGTCCCTTTTGATGAGAAGAATAAGCCTCATTCTGATTCAACAGCAGAGATCAAAG
AAAAGACTTCTGTTTTCTGGCCACCAGATATATGTTATCTGTGCTTAAAGAATTGAAAAACACACATCAA
AGGAGAATTTTCTTGGAAAGAGAGGGTTCAAGCATCACTGTTAGGTGTGCTGGAATCCTTTCCCGAGTCA
GTACTGCTTTCTAGAAGAAAACCGGGGAGATCTATTTGGAATGTATCTAACTCCAAAGAAACCATCAGAG
GTAACAGTAGAGACGGGGTTTCACCATGTTGGCCAGACTGGTCTTGAACTCCCGACCTCGTGATTCGCCC
GCCTCGGCCTCCCAAAGTGCTGGGATTACAGGTGTGAGACACCACGCCCGGCGGATAGAGAGAATTTTGA
CAG*GTGAGGAGGTATTCCAATGCAAAAGAATAATAGGAGCAAAAGCACAGTGGTGAGAAATTGGAGGGGA*
ACTGTGAAAATTGCCACATAGATTAGAGGCAGGAAAATAAAGGACGGCTAAGTTTATATAGTGAACAGTG
*AGC*AAGCTTGAATTC

>circANRIL-BoxB, 928 bp without restriction sites, 648 bp exonic sequence incl. BoxB

GAATTCGCGGCCGC*CAATGTATTTCTCTGCTTCATACACTATCCAAGGTGATAAAAGTGTTTATGAAGAG*
GGATTAGGAAATATCTGCTGTGTTTAGGTCATAGTCCAACCAAAGGATTAATTAACATTTGCCTCTTTTT
*TCTCCTACATCCAG*TGTCCCTTTTGATGAGAAGAATAAGCCTCATTCTGATTCAACAGCAGAGATCAAAG
AAAAGACTTCTGTTTTCTGGCCACCAGATATATGTTATCTGTGCTTAAAGAATTGAAAAACACACATCAA
AGGAGAATTTTCTTGGAAAGAGAGGGTTCAAGCATCACTGTTAGGTGTGCTGGAATCCTTTCCATGGCAC
CATGTGGAATTCTCCGGAAAGCTTTCGCGACTCGAGACTAGTTCTAGATGATCACCCGAGGGGCCCTGAA
GAAGGGCCCCTCGACTAAGTCCAACTACTAAACTGGGCCCTGAAGAAGGGCCCATATAGGGCCCTGAAGA
AGGGCCCTATCGAGGATATTATCTCGACTAAGTCCAACTACTAAACTGGGCCCTGAAGAAGGGCCCATAT
AGGGCCCTGAAGAAGGGCCCCCCGAGTCAGTACTGCTTTCTAGAAGAAAACCGGGGAGATCTATTTGGAA
TGTATCTAACTCCAAAGAAACCATCAGAGGTAACAGTAGAGACGGGGTTTCACCATGTTGGCCAGACTGG
TCTTGAACTCCCGACCTCGTGATTCGCCCGCCTCGGCCTCCCAAAGTGCTGGGATTACAGGTGTGAGACA
CCACGCCCGGCGGATAGAGAGAATTTTGACAG*GTGAGGAGGTATTCCAATGCAAAAGAATAATAGGAGCA*
AAAGCACAGTGGTGAGAAATTGGAGGGGAACTGTGAAAATTGCCACATAGATTAGAGGCAGGAAAATAAA
*GGACGGCTAAGTTTATATAGTGAACAGTGAGC*AAGCTTGAATTC

>BoxB, 170 bp without restriction sites

AAGCTTGGGCCCTGAAGAAGGGCCCCTCGACTAAGTCCAACTACTAAACTGGGCCCTGAAGAAGGGCCCA
TATAGGGCCCTGAAGAAGGGCCCTATCGAGGATATTATCTCGACTAAGTCCAACTACTAAACTGGGCCCT
GAAGAAGGGCCCATATAGGGCCCTGAAGAAGGGCCCGTCGACAAGCTT

>circHPRT1(ex2-3-4-5-6), 738 bp without restriction sites, 458 bp exonic sequence

GAATTCGCGGCCGC*ACCTACAAAGTTAACAGTTACTAATATCATCTTACACCTAAATTTCTCTGATAGAC*
TAAGGTTATTTTTTAACATCTTAATCCAATCAAATGTTTGTATCCTGTAATGCTCTCATTGAAACAGCTA
*TATTTCTTTTTCAG*ATTAGTGATGATGAACCAGGTTATGACCTTGATTTATTTTGCATACCTAATCATTA
TGCTGAGGATTTGGAAAGGGTGTTTATTCCTCATGGACTAATTATGGACAGGACTGAACGTCTTGCTCGA
GATGTGATGAAGGAGATGGGAGGCCATCACATTGTAGCCCTCTGTGTGCTCAAGGGGGGCTATAAATTCT
TTGCTGACCTGCTGGATTACATCAAAGCACTGAATAGAAATAGTGATAGATCCATTCCTATGACTGTAGA
TTTTATCAGACTGAAGAGCTATTGTAATGACCAGTCAACAGGGGACATAAAAGTAATTGGTGGAGATGAT
CTCTCAACTTTAACTGGAAAGAATGTCTTGATTGTGGAAGATATAATTGACACTGGCAAAACAATGCAGA
CTTTGCTTTCCTTGGTCAGGCAGTATAATCCAAAGATGGTCAAGGTCGCAAG*GTATGTATGACATTTTGA*
CACAGAATATTTTCCTCATTTGAAGGGGGATTAAGTGATTGCTTCTTTTTAAGGATAAATGTTTTCAACT
*GTCATTTTATCTTCGAAAAGTAATGTAATCTCATATAAGACTTAAGATATAA*AAGCTTGAATTC

DNA sequences for overexpression of circular RNA were cloned into the bicistronic pBI-CMV2 vector (Clontech). GAATTC (dark blue)- EcoRI restriction site, GCGGCCGC (grey) – NotI restriction site, AAGCTT (light blue)- HindIII restriction site, ACGTGGACGT- exonic sequence, exon-exon boundary, *ACGT*- intronic sequence, *ACGT*- BoxB sequence.

Supplementary Table 2. Sequence validation of ANRIL genomic knockout in HEK-293 cells. Related to Figure 3.

>9p21 heterozygous knockout cell line, 202 bp PCR product

GGCAGGAGGATCTGGGGCATATGTTTAAATCAGGCATTGGCATCTGTACTAGAATTGAAAGGTGGAACTA
TTATTGTGTTCTATTTATTGAGTATAGGTATACCATATACCTATCATGGCAAGGGTGATGGGTAGATGGG
TAGAGGTTGCTCAAAAAGAAACACAAAATATATCCTCTTTTAAGGATCCTGATGCTTTGCTC

>9p21 homozygous knockout cell line, 226 bp PCR product

GGCAGGAGGATCTGGGGCATATGTTTAAATCAGGCATTGGCATCTGTACTAGAATTGAAAGGTGGAACTA
TTATTGTGTTCTATTTATTGAGTATAGGTATACCATATACTATAAGTATAGGTATAGTATACCATACCCT
TGGCAAGGGTGATGGGTAGATGGGTAGAGGTTGCTCAAAAAGAAACACAAAATATATCCTCTTTTAAGGA
TCCTGATGCTTTGCTC

DNA sequences from HEK-293 cell lines with heterozygous or homozygous knockout of 9p21 region. ACGTACGT - genomic sequence, ACGTACGT - primers (see also Supplementary Table 7).

Supplementary Table 3. *circANRIL*-bound proteins identified by label-free mass-spectrometric quantification (LFQ).

Proteins	MS/MS Count	LFQ circANRIL+BoxB (log intensity)	LFQ circANRIL (log intensity)	Difference (Δlog)	-log(p)*
NOP14	145	29.07	26.86	2.21	2.09
PES1	117	28.03	25.99	2.04	1.38
SDAD1	27	28.10	26.52	1.58	1.61
LYAR	372	26.95	25.66	1.29	1.67
EIF1AX;EIF1AY	19	27.85	26.67	1.18	2.36
NOLC1	338	30.32	29.18	1.14	2.85
FRG1;FRG1B	57	28.07	26.94	1.12	1.44
DDX54	203	26.13	25.08	1.05	1.48
BMS1	159	26.85	25.92	0.93	2.00
SURF6	219	26.89	26.01	0.88	1.41
ARL6IP4	218	32.07	31.19	0.88	2.95
RFC1	68	31.50	30.63	0.87	2.74
NAT10	356	30.25	29.40	0.86	1.36
NOM1	121	28.78	27.95	0.83	2.16
SLC16A1	35	28.30	27.48	0.82	1.33
MAZ	52	28.43	27.63	0.81	2.88
AKAP17A	232	28.44	27.66	0.78	1.53
UBTF	172	29.59	28.85	0.74	2.16
SF3B2	82	28.67	27.96	0.71	2.30
RPL22	94	30.25	29.67	0.58	2.92
HSPA8	339	30.42	29.90	0.53	2.93
THRAP3	290	30.19	29.68	0.51	1.51
RRP1	143	29.63	29.13	0.50	2.18
RPL36A	84	30.66	30.18	0.48	1.43
PRPF38B	131	29.11	28.65	0.47	2.26
ESF1	112	28.19	27.73	0.46	2.34
PRPF40A	320	30.94	30.49	0.45	1.89
PGAM5	170	30.90	30.46	0.43	1.76
RPL19	241	32.77	32.35	0.42	1.82
PRPF3	164	29.44	29.07	0.37	2.29
ZNF512	88	28.24	27.88	0.36	1.37
RPL13	301	32.50	32.23	0.26	2.09
NOP14	145	29.07	26.86	2.21	2.09
HADHB	24	28,12	25,93	2.04	1.38

* proteins with significant binding ($p < 0.05$; e.g. (-log (p) > 1.3) are given.

Supplementary Table 4 *De novo* translated proteins in *circANRIL* overexpressing compared to vector control (CTR) HEK-293 cells.

Gene name	Protein name	Ratio 48h/24h (circANRIL/CTR)
SNRPD1	Small nuclear ribonucleoprotein Sm D1	-1.56
EPPK1	Epiplakin	-1.41
EIF1;EIF1B	Eukaryotic translation initiation factor 1;Eukaryotic translation initiation factor 1b	-1.41
CARM1	Histone-arginine methyltransferase CARM1	-1.40
HSPA4L	Heat shock 70 kDa protein 4L	-1.37
TBCE	Tubulin-specific chaperone E	-1.35
CNOT1	CCR4-NOT transcription complex subunit 1	-1.34
KPNA2	Importin subunit alpha-1	-1.33
AIMP1	Aminoacyl tRNA synthase complex-interacting multifunctional protein 1;Endothelial monocyte-activating polypeptide 2	-1.32
BOP1;KM-PA-2	Ribosome biogenesis protein BOP1	-1.32
PSIP1	PC4 and SFRS1-interacting protein	-1.32
GCLM	Glutamate--cysteine ligase regulatory subunit	-1.28
HEATR1	HEAT repeat-containing protein 1	-1.28
TRMT112	tRNA methyltransferase 112 homolog	-1.27
THOC2	THO complex subunit 2	-1.26
CBX5	Chromobox protein homolog 5	-1.26
PPIH	Peptidyl-prolyl cis-trans isomerase H;Peptidyl-prolyl cis-trans isomerase	-1.26
IMPDH1	Inosine-5-monophosphate dehydrogenase 1;Inosine-5-monophosphate dehydrogenase	-1.26
TIAL1	Nucleolysin TIAR	-1.26
EIF4G2	Eukaryotic translation initiation factor 4 gamma 2	-1.26
NOP56	Nucleolar protein 56	-1.25
KRR1	KRR1 small subunit processome component homolog	-1.23
DKC1	H/ACA ribonucleoprotein complex subunit 4	-1.22
HSPA14	Heat shock 70 kDa protein 14	-1.22
EIF5	Eukaryotic translation initiation factor 5	-1.21
ARPC1A	Actin-related protein 2/3 complex subunit 1A	-1.21
MRPL38	39S ribosomal protein L38, mitochondrial	-1.21
H2AFY2	Core histone macro-H2A.2	-1.20
TOP2A	DNA topoisomerase 2-alpha	-1.20
VPS29	Vacuolar protein sorting-associated protein 29	-1.20
CDV3	Protein CDV3 homolog	-1.20
SCAF4	Splicing factor, arginine/serine-rich 15	-1.19
CFL2	Cofilin-2	-1.19
NUP93	Nuclear pore complex protein Nup93	-1.19
PPIF	Peptidyl-prolyl cis-trans isomerase F, mitochondrial	-1.19
RPL35	60S ribosomal protein L35	-1.19
SKIV2L2	Superkiller viralicidic activity 2-like 2	-1.19
DNAJC8	DnaJ homolog subfamily C member 8	-1.19

UGDH	UDP-glucose 6-dehydrogenase	-1.18
HUWE1	E3 ubiquitin-protein ligase HUWE1	-1.18
EEF1A2	Elongation factor 1-alpha 2	-1.18
TOP1	DNA topoisomerase 1	-1.18
EMD	Emerin	-1.18
METAP2	Methionine aminopeptidase 2;Methionine aminopeptidase	-1.18
DIABLO	Diablo homolog, mitochondrial	-1.17
WBP11	WW domain-binding protein 11	-1.17
ACTA1;ACTC1; ACTG2;ACTA2	Actin, alpha skeletal muscle;Actin, alpha cardiac muscle 1;Actin, gamma-enteric smooth muscle;Actin, aortic smooth muscle	-1.17
RFC5	Replication factor C subunit 5	-1.17
KPNA1	Importin subunit alpha-5	-1.17
RRP1B	Ribosomal RNA processing protein 1 homolog B	-1.16
PACSIN2	Protein kinase C and casein kinase substrate in neurons protein 2	-1.16
PPA2	Inorganic pyrophosphatase 2, mitochondrial	-1.16
DDX23	Probable ATP-dependent RNA helicase DDX23	-1.16
DRG1	Developmentally-regulated GTP-binding protein 1	-1.16
PPP1R2;PPP1R2 P3	Protein phosphatase inhibitor 2;Putative protein phosphatase inhibitor 2-like protein 3	-1.16
CBX3	Chromobox protein homolog 3	-1.16
TPR	Nucleoprotein TPR	-1.16
EIF2S3;EIF2S3L	Eukaryotic translation initiation factor 2 subunit 3;Putative eukaryotic translation initiation factor 2 subunit 3-like protein	-1.15
RRP12	RRP12-like protein	-1.15
EIF3K	Eukaryotic translation initiation factor 3 subunit K	-1.15
PLEC	Plectin	-1.15
NUP205	Nuclear pore complex protein Nup205	-1.15
SCFD1	Sec1 family domain-containing protein 1	-1.15
SNRPA1	U2 small nuclear ribonucleoprotein A	-1.15
AKAP12	A-kinase anchor protein 12	-1.15
BZW2	Basic leucine zipper and W2 domain-containing protein 2	-1.15
EIF4G1	Eukaryotic translation initiation factor 4 gamma 1	-1.15
PRPF6	Pre-mRNA-processing factor 6	-1.14
BZW1	Basic leucine zipper and W2 domain-containing protein 1	-1.14
MST4	Serine/threonine-protein kinase MST4	-1.14
NHP2L1	NHP2-like protein 1;NHP2-like protein 1, N-terminally processed	-1.14
RPL29	60S ribosomal protein L29	-1.14
GDI1	Rab GDP dissociation inhibitor alpha	-1.13
HSP90AB1	Heat shock protein HSP 90-beta	-1.13
SNRPD2	Small nuclear ribonucleoprotein Sm D2	-1.13
DFFA	DNA fragmentation factor subunit alpha	-1.13

NUTF2	Nuclear transport factor 2	-1.13
SUPT5H	Transcription elongation factor SPT5	-1.13
ENO2	Gamma-enolase;Enolase	-1.13
GFPT1	Glutamine--fructose-6-phosphate aminotransferase [isomerizing] 1	-1.13
RPL30	60S ribosomal protein L30	-1.13
MTPN	Myotrophin	-1.13
EIF3D	Eukaryotic translation initiation factor 3 subunit D	-1.13
UBQLN2	Ubiquilin-2	-1.12
RBM39	RNA-binding protein 39	-1.12
VCL	Vinculin	-1.12
DIS3	Exosome complex exonuclease RRP44	-1.12
IPO9	Importin-9	-1.12
SRRT	Serrate RNA effector molecule homolog	-1.12
COPE	Coatomer subunit epsilon	-1.12
U2AF2	Splicing factor U2AF 65 kDa subunit	-1.12
MLEC	Malectin	-1.12
RPS20	40S ribosomal protein S20	-1.12
PRPS1	Ribose-phosphate pyrophosphokinase 1;Ribose-phosphate pyrophosphokinase	-1.12
SNRNP70	U1 small nuclear ribonucleoprotein 70 kDa	-1.12
MTA2;DKFZp686F2281	Metastasis-associated protein MTA2	-1.12
GFM1	Elongation factor G, mitochondrial	-1.12
TOMM22	Mitochondrial import receptor subunit TOM22 homolog	-1.12
TMPO	Lamina-associated polypeptide 2, isoform alpha;Thymopoietin;Thymopentin	-1.12
PSME3	Proteasome activator complex subunit 3	-1.11
RRS1	Ribosome biogenesis regulatory protein homolog	-1.11
DUT	Deoxyuridine 5-triphosphate nucleotidohydrolase, mitochondrial	-1.11
UCHL5	Ubiquitin carboxyl-terminal hydrolase isozyme L5	-1.11
BANF1	Barrier-to-autointegration factor;Barrier-to-autointegration factor, N-terminally processed	1.10
CLTB	Clathrin light chain B	1.10
VPS35	Vacuolar protein sorting-associated protein 35	1.10
MDH1	Malate dehydrogenase, cytoplasmic;Malate dehydrogenase	1.10
TSN	Translin	1.11
DNM2	Dynamin-2	1.11
SKP1	S-phase kinase-associated protein 1	1.11
TTLL12	Tubulin--tyrosine ligase-like protein 12	1.11
PAFAH1B3	Platelet-activating factor acetylhydrolase IB subunit gamma	1.11
PSMB5	Proteasome subunit beta type-5	1.11
ANXA7	Annexin A7;Annexin	1.11
HNRNPUL1	Heterogeneous nuclear ribonucleoprotein U-like protein	1.11

	1	
EDC4	Enhancer of mRNA-decapping protein 4	1.11
HIST1H1C	Histone H1.2	1.11
PFKP	6-phosphofructokinase type C	1.11
G6PD	Glucose-6-phosphate 1-dehydrogenase	1.11
HSPA1A	Heat shock 70 kDa protein 1A/1B	1.11
BID	BH3-interacting domain death agonist;BH3-interacting domain death agonist p15;BH3-interacting domain death agonist p13;BH3-interacting domain death agonist p11	1.12
FUBP3	Far upstream element-binding protein 3	1.12
ACAT2	Acetyl-CoA acetyltransferase, cytosolic	1.12
FKBP3	Peptidyl-prolyl cis-trans isomerase FKBP3	1.12
RPL19	60S ribosomal protein L19	1.12
NDUFS3	NADH dehydrogenase [ubiquinone] iron-sulfur protein 3, mitochondrial	1.12
MAPK1	Mitogen-activated protein kinase 1	1.12
CHORDC1	Cysteine and histidine-rich domain-containing protein 1	1.12
GLOD4	Glyoxalase domain-containing protein 4	1.12
PPIB	Peptidyl-prolyl cis-trans isomerase B	1.12
DDX1	ATP-dependent RNA helicase DDX1	1.12
RAB2A;RAB2B	Ras-related protein Rab-2A;Ras-related protein Rab-2B	1.12
NNT	NAD(P) transhydrogenase, mitochondrial	1.13
HAT1	Histone acetyltransferase type B catalytic subunit	1.13
APRT	Adenine phosphoribosyltransferase	1.13
ASNA1	ATPase ASNA1	1.13
TKT	Transketolase	1.14
CLUH	Clustered mitochondria protein homolog	1.14
EPB41L3	Band 4.1-like protein 3;Band 4.1-like protein 3, N-terminally processed	1.14
ECH1	Delta(3,5)-Delta(2,4)-dienoyl-CoA isomerase, mitochondrial	1.14
PDLIM1	PDZ and LIM domain protein 1	1.14
PHF6	PHD finger protein 6	1.14
SNRPB2	U2 small nuclear ribonucleoprotein B	1.14
MCMBP	Mini-chromosome maintenance complex-binding protein	1.14
IARS	Isoleucine--tRNA ligase, cytoplasmic	1.15
THOC3	THO complex subunit 3	1.15
GSTM3	Glutathione S-transferase Mu 3	1.15
CBR1	Carbonyl reductase [NADPH] 1	1.15
NDUFS2	NADH dehydrogenase [ubiquinone] iron-sulfur protein 2, mitochondrial	1.15
TCERG1	Transcription elongation regulator 1	1.15
PHPT1	14 kDa phosphohistidine phosphatase	1.15
RCN2	Reticulocalbin-2	1.15
NASP	Nuclear autoantigenic sperm protein	1.16
ECI1;DCI	Enoyl-CoA delta isomerase 1, mitochondrial	1.16

GPD2	Glycerol-3-phosphate dehydrogenase, mitochondrial	1.16
MCM2	DNA replication licensing factor MCM2	1.16
ALDH9A1	4-trimethylaminobutyraldehyde dehydrogenase	1.16
PCBP2;PCBP3	Poly(rC)-binding protein 2;Poly(rC)-binding protein 3	1.16
PSMB6	Proteasome subunit beta type-6	1.17
SEC24C	Protein transport protein Sec24C	1.17
DCTN1	Dynactin subunit 1	1.17
UAP1	UDP-N-acetylhexosamine pyrophosphorylase;UDP-N-acetylgalactosamine pyrophosphorylase;UDP-N-acetylglucosamine pyrophosphorylase	1.17
APMAP	Adipocyte plasma membrane-associated protein	1.17
GOT1	Aspartate aminotransferase, cytoplasmic;Aspartate aminotransferase	1.17
MCM5	DNA replication licensing factor MCM5	1.17
MCM3	DNA replication licensing factor MCM3	1.18
ACO2	Aconitate hydratase, mitochondrial	1.18
RHOA;RHOC	Transforming protein RhoA;Rho-related GTP-binding protein RhoC	1.19
MRTO4	mRNA turnover protein 4 homolog	1.19
TOMM40	Mitochondrial import receptor subunit TOM40 homolog	1.19
ATP6V1B2	V-type proton ATPase subunit B, brain isoform	1.20
ANXA6	Annexin A6;Annexin	1.20
COPS2	COP9 signalosome complex subunit 2	1.20
MRI1	Methylthioribose-1-phosphate isomerase	1.20
DPYSL2	Dihydropyrimidinase-related protein 2	1.21
BCAP31	B-cell receptor-associated protein 31	1.21
PGP	Phosphoglycolate phosphatase	1.21
KIAA1279	KIF1-binding protein	1.21
PSME1	Proteasome activator complex subunit 1	1.23
CPVL	Probable serine carboxypeptidase CPVL	1.23
IDE	Insulin-degrading enzyme	1.23
GPS1	COP9 signalosome complex subunit 1	1.23
PGD	6-phosphogluconate dehydrogenase, decarboxylating	1.24
IDH1	Isocitrate dehydrogenase [NADP] cytoplasmic	1.24
PRDX2	Peroxiredoxin-2	1.25
HEXB	Beta-hexosaminidase subunit beta;Beta-hexosaminidase subunit beta chain B;Beta-hexosaminidase subunit beta chain A	1.25
RFC2	Replication factor C subunit 2	1.27
CAPNS1	Calpain small subunit 1	1.28
NEFL	Neurofilament light polypeptide	1.28
GLUD1;GLUD2	Glutamate dehydrogenase 1, mitochondrial;Glutamate dehydrogenase;Glutamate dehydrogenase 2, mitochondrial	1.29
TPP2	Tripeptidyl-peptidase 2	1.30
APEH	Acylamino-acid-releasing enzyme	1.30
MRPS22	28S ribosomal protein S22, mitochondrial	1.30

BPNT1	3(2),5-bisphosphate nucleotidase 1	1.32
CUL4B	Cullin-4B	1.36
COMT	Catechol O-methyltransferase	1.43
BLVRA	Biliverdin reductase A	1.68

De novo translated proteins with expression changes +/- 10% within 24 h in *circANRIL* overexpressing relative to control cells (n=197) are listed.

Supplementary Table 5 Upstream regulator analysis of differentially *de novo* translated proteins in *circANRIL* overexpressing and vector control HEK-293 cells.

Transcription regulator	p-value
HNF4A	2.43×10^{-8}
MYC	3.19×10^{-8}
TP53	3.45×10^{-7}
NFE2L2	8.14×10^{-7}
KDM5B	4.84×10^{-6}
E2F1	1.15×10^{-5}
E2F4	8.11×10^{-5}
MYCN	1.67×10^{-4}

De novo translated proteins with expression changes +/- 10% within 24 h in *circANRIL* overexpressing relative to control cells (n=197) were included in the analysis.

Supplementary Table 6 Upstream regulator analysis of differentially regulated genes in *circANRIL* overexpressing and vector control HEK-293 cells.

**Transcription regulator**	**p-value**
TP53	8.53×10^{-16}
MYC	8.27×10^{-09}
NUPR1	2.67×10^{-08}
TP63	1.35×10^{-07}
E2F1	5.23×10^{-07}
HNF4A	6.11×10^{-07}
HIF1A	9.38×10^{-07}
EPAS1	1.40×10^{-05}

Genes with expression fold changes of < 0.5 and > 2 in *circANRIL* overexpressing cell lines (n=3; n= 1809) compared to vector control cell lines (n=3) were included in the analysis.

Supplementary Table 7 Sequence alignment of *circANRIL* and precursor S45S5 rRNA (NM_046235) using the BLASTn algorithm.

Motif	Sequence alignment	bp (circANRIL)
M1	circANRIL 81 CAGATATATGTTATCTGTGCTT 102<br>|||| || || | | ||||||| RNA45S5 12545 CAGACATTTGGTGTATGTGCTT 12566	81-102
M2	circANRIL 144 AAGAGAGGGTTCAAG 158<br>||||||| ||||||| RNA45S5 8319 AAGAGAGAGTTCAAG 8333	144-158
M3	circANRIL 276 GAGACGGGGTTTCACCATGTTGGCCAGAC 304<br>|||| |||||| | || | |||||||| RNA45S5 6509 GAGAGGGGGTTGCCTCAGGCCGGCCAGAC 6481	276-304
M4	circANRIL 332 CGCCCGCCTCGGCCTCCCAAAGTGCTGGGA 361<br>|||| ||| | || |||||| ||||||| RNA45S5 1713 CGCCGGCCGCCGCGTCCCAACCCGCTGGGA 1684	332-361
M5	circANRIL 381 CGCCCGGCGGATAGAGA 397<br>|||||||| || ||||| RNA45S5 6965 CGCCCGGCTGAGAGAGA 6981	381-397

Supplementary Table 8. Plasmid sequences for PES1 wildtype (PES1-WT), PES1Δ1-54 and PES1Δ446-588 overexpression.

>PES1-WT, 1790 bp exonic sequence

```
GGATCCGTCGCCACCATGGGAGGCCTTGAGAAGAAGTATGAACGAGGCTCGGCCACCAACTACATCA
CCCGGAACAAAGCCCGGAAGAAGCTCCAGCTGAGCTTGGCTGACTTTAGGCGGCTGTGCATTCTGAAGGG
CATTTATCCCCATGAACCCAAACACAAGAAGAAGGTTAACAAGGGTTCTACAGCAGCCCGAACGTTTTAC
CTTATCAAAGACATCAGGTTTCTCCTCCACGAACCCATTGTCAACAAGTTCCGTGAATACAAGGTGTTCG
TCCGGAAGCTCCGGAAGGCTTATGGGAAGAGCGAGTGGAACACTGTAGAGCGTTTAAAGGACAATAAGCC
CAACTACAAACTCGACCACATCATCAAGGAACGGTATCCCACGTTCATCGATGCCCTGCGGGACCTGGAC
GATGCCCTCTCCATGTGCTTCCTGTTTTCCACCTTCCCGCGGACTGGCAAGTGCCACGTGCAGACCATTC
AGCTGTGCCGCCGGCTCACTGTGGAGTTCATGCACTACATTATCGCTGCCCGTGCCCTGCGCAAGGTCTT
CCTGTCCATCAAAGGCATTTACTACCAGGCCGAGGTACTGGGGCAGCCCATCGTGTGGATCACTCCCTAT
GCCTTCTCCCATGACCACCCGACAGACGTGGACTACAGGGTCATGGCCACCTTCACCGAGTTCTACACCA
CGCTGCTGGGCTTTGTCAACTTCCGCCTTTACCAGTTGCTCAACCTCCACTATCCCCCGAAGCTCGAGGG
TCAGGCCCAAGCAGAGGCAAAGGCCGGTGAGGGCACCTACGCGTTGGACTCCGAGAGTTGTATGGAGAAA
CTGGCAGCCCTCAGTGCCAGCCTGGCCCGCGTGGTGGTGCCTGCCACAGAGGAGGAGGCCGAGGTGGATG
AGTTTCCCACCGATGGGGAGATGTCAGCGCAGGAGGAAGACCGCAGGAAGGAGCTGGAGGCGCAGGAGAA
GCACAAGAAGCTTTTTGAGGGCCTGAAGTTCTTCCTGAACCGAGAGGTGCCCCGTGAGGCCCTGGCCTTC
ATCATCAGGAGTTTTGGTGGGGAAGTGTCCTGGGACAAATCTTTGTGCATTGGGGCCACCTATGACGTCA
CAGACTCCCGCATCACCCATCAGATTGTCGACCGGCCTGGGCAGCAGACCTCAGTCATTGGCAGGTGCTA
CGTGCAGCCCCAGTGGGTGTTTGACTCAGTGAACGCCAGGCTCCTTCTCCCCGTGGCAGAGTACTTCTCT
GGGGTGCAGCTGCCCCCACACCTTTCACCCTTTGTGACCGAGAAGGAAGGAGATTACGTTCCACCTGAGA
AGCTGAAGCTGCTGGCTCTGCAGCGGGGAGAGGACCCAGGAAACCTGAATGAGTCAGAAGAGGAGGAGGA
AGAGGACGACAACAACGAAGGTGATGGTGATGAAGAGGGAGAAAATGAGGAGGAGGAGGAAGATGCAGAG
GCTGGTTCAGAAAAGGAGGAAGAGGCCCGGCTGGCAGCCCTGGAAGAGCAGAGGATGGAGGGGAAGAAGC
CCAGGGTGATGGCAGGCACCTTGAAGCTGGAGGATAAGCAGCGGCTGGCCCAGGAGGAGGAGAGTGAGGC
CAAGCGCCTGGCCATTATGATGATGAAGAAGCGGGAGAAGTACCTGTACCAGAAGATCATGTTTGGCAAG
AGGCGAAAAATCCGAGAGGCCAACAAGCTGGCGGAGAAGCGGAAAGCCCACGATGAGGCGGTGAGGTCTG
AGAAGAAGGCCAAGAAGGCAAGGCCGGAGGGGGGGCCACCGGTCTGAGCGGCCGC
```

>PES1□1-54, 1605 bp exonic sequence

```
GGATCCGTCGCCACCATGAACAAGGGTTCTACAGCAGCCCGAACGTTTTACCTTATCAAAGACATCAGGT
TTCTCCTCCACGAACCCATTGTCAACAAGTTCCGTGAATACAAGGTGTTCGTCCGGAAGCTCCGGAAGGC
TTATGGGAAGAGCGAGTGGAACACTGTAGAGCGTTTAAAGGACAATAAGCCCAACTACAAACTCGACCAC
ATCATCAAGGAACGGTATCCCACGTTCATCGATGCCCTGCGGGACCTGGACGATGCCCTCTCCATGTGCT
TCCTGTTTTCCACCTTCCCGCGGACTGGCAAGTGCCACGTGCAGACCATTCAGCTGTGCCGCCGGCTCAC
TGTGGAGTTCATGCACTACATTATCGCTGCCCGTGCCCTGCGCAAGGTCTTCCTGTCCATCAAAGGCATT
TACTACCAGGCCGAGGTACTGGGGCAGCCCATCGTGTGGATCACTCCCTATGCCTTCTCCCATGACCACC
CGACAGACGTGGACTACAGGGTCATGGCCACCTTCACCGAGTTCTACACCACGCTGCTGGGCTTTGTCAA
CTTCCGCCTTTACCAGTTGCTCAACCTCCACTATCCCCCGAAGCTCGAGGGTCAGGCCCAAGCAGAGGCA
AAGGCCGGTGAGGGCACCTACGCGTTGGACTCCGAGAGTTGTATGGAGAAACTGGCAGCCCTCAGTGCCA
GCCTGGCCCGCGTGGTGGTGCCTGCCACAGAGGAGGAGGCCGAGGTGGATGAGTTTCCCACCGATGGGGA
GATGTCAGCGCAGGAGGAAGACCGCAGGAAGGAGCTGGAGGCGCAGGAGAAGCACAAGAAGCTTTTTGAG
GGCCTGAAGTTCTTCCTGAACCGAGAGGTGCCCCGTGAGGCCCTGGCCTTCATCATCAGGAGTTTTGGTG
GGGAAGTGTCCTGGGACAAATCTTTGTGCATTGGGGCCACCTATGACGTCACAGACTCCCGCATCACCCA
TCAGATTGTCGACCGGCCTGGGCAGCAGACCTCAGTCATTGGCAGGTGCTACGTGCAGCCCCAGTGGGTG
TTTGACTCAGTGAACGCCAGGCTCCTTCTCCCCGTGGCAGAGTACTTCTCTGGGGTGCAGCTGCCCCCAC
ACCTTTCACCCTTTGTGACCGAGAAGGAAGGAGATTACGTTCCACCTGAGAAGCTGAAGCTGCTGGCTCT
GCAGCGGGGAGAGGACCCAGGAAACCTGAATGAGTCAGAAGAGGAGGAGGAAGAGGACGACAACAACGAA
GGTGATGGTGATGAAGAGGGAGAAAATGAGGAGGAGGAGGAAGATGCAGAGGCTGGTTCAGAAAAGGAGG
AAGAGGCCCGGCTGGCAGCCCTGGAAGAGCAGAGGATGGAGGGGAAGAAGCCCAGGGTGATGGCAGGCAC
CTTGAAGCTGGAGGATAAGCAGCGGCTGGCCCAGGAGGAGGAGAGTGAGGCCAAGCGCCTGGCCATTATG
ATGATGAAGAAGCGGGAGAAGTACCTGTACCAGAAGATCATGTTTGGCAAGAGGCGAAAAATCCGAGAGG
CCAACAAGCTGGCGGAGAAGCGGAAAGCCCACGATGAGGCGGTGAGGTCTGAGAAGAAGGCCAAGAAGGC
AAGGCCGGAGGGGGGGCCACCGGTCTGAGCGGCCGC
```

>PES1Δ446-588, 1335 bp exonic sequence

```
GGATCCGTCGCCACCATGGGAGGCCTTGAGAAGAAGAAGTATGAACGAGGCTCGGCCACCAACTACATCAC
CCGGAACAAAGCCCGGAAGAAGCTCCAGCTGAGCTTGGCTGACTTTAGGCGGCTGTGCATTCTGAAGGGCA
TTTATCCCCATGAACCCAAACACAAGAAGAAGGTTAACAAGGGTTCTACAGCAGCCCGAACGTTTTACCTT
ATCAAAGACATCAGGTTTCTCCTCCACGAACCCATTGTCAACAAGTTCCGTGAATACAAGGTGTTCGTCCG
GAAGCTCCGGAAGGCTTATGGGAAGAGCGAGTGGAACACTGTAGAGCGTTTAAAGGACAATAAGCCCAACT
ACAAACTCGACCACATCATCAAGGAACGGTATCCCACGTTCATCGATGCCCTGCGGGACCTGGACGATGCC
CTCTCCATGTGCTTCCTGTTTTCCACCTTCCCGCGGACTGGCAAGTGCCACGTGCAGACCATTCAGCTGTG
CCGCCGGCTCACTGTGGAGTTCATGCACTACATTATCGCTGCCCGTGCCCTGCGCAAGGTCTTCCTGTCCA
TCAAAGGCATTTACTACCAGGCCGAGGTACTGGGGCAGCCCATCGTGTGGATCACTCCCTATGCCTTCTCC
CATGACCACCCGACAGACGTGGACTACAGGGTCATGGCCACCTTCACCGAGTTCTACACCACGCTGCTGGG
CTTTGTCAACTTCCGCCTTTACCAGTTGCTCAACCTCCACTATCCCCCGAAGCTCGAGGGTCAGGCCCAAG
CAGAGGCAAAGGCCGGTGAGGGCACCTACGCGTTGGACTCCGAGAGTTGTATGGAGAAACTGGCAGCCCTC
AGTGCCAGCCTGGCCCGCGTGGTGGTGCCTGCCACAGAGGAGGAGGCCGAGGTGGATGAGTTTCCCACCGA
TGGGGAGATGTCAGCGCAGGAGGAAGACCGCAGGAAGGAGCTGGAGGCGCAGGAGAAGCACAAGAAGCTTT
TTGAGGGCCTGAAGTTCTTCCTGAACCGAGAGGTGCCCCGTGAGGCCCTGGCCTTCATCATCAGGAGTTTT
GGTGGGGAAGTGTCCTGGGACAAATCTTTGTGCATTGGGGCCACCTATGACGTCACAGACTCCCGCATCAC
CCATCAGATTGTCGACCGGCCTGGGCAGCAGACCTCAGTCATTGGCAGGTGCTACGTGCAGCCCCAGTGGG
TGTTTGACTCAGTGAACGCCAGGCTCCTTCTCCCCGTGGCAGAGTACTTCTCTGGGGTGCAGCTGCCCCCA
CACCTTTCACCCTTTGTGACCGAGAAGGAAGGAGATTACGTTCCACCTGAGAAGCTGAAGCTGCTGGCTCT
GGGGGGGCCACCGGTCTGAGCGGCCGC
```

DNA sequences for overexpression of PES1 wildtype (PES1-WT) and truncated PES1 (PES1□1-54 and PES1□446-588, respectively) were cloned in the pmCherry-N1 vector (Clontech). GGATCC (dark blue)- BamHI restriction site, ACCGGT (grey) – AgeI restriction site, GCGGCCGC (light blue)- NotI restriction site, ACGTACGT - exonic sequence, <u>ACGTACGT</u>- tag-linking sequence.

Supplementary Table 9 Primers and probes for PCR and qPCR.

**Transcript/ NM number**	**Primer/ probe**	**Sequence**
circANRIL (*ex7-5*)	5’-primer	5’-GCTGGGATTACAGGTGTGAGACACC-3’
	3’-primer	5’-GAATCAGAATGAGGCTTATTCTTCTCATC-3’
	probe	5’F-CCCGGCGGATAGAGAGAATTTTGACAGTGTC-3‘T
lin+circANRIL (*ex6-7*)	5’-primer	5’-GGGAGATCTATTTGGAATGTATCTAACTCC-3’
	3’-primer	5’-GAGAGTTCAAGACCAGTCTGGCC-3’
	probe	5’F-CCATCAGAGGTAACAGTAGAGACGGGGTTTCACC-3‘T
circHPRT1 (*ex6-2*)	5’-primer	5’-GGTCAGGCAGTATAATCCAAAGATGG-3’
NM_000194	3’-primer	5’-CAAATCCTCAGCATAATGATTAGGTATGC-3’
	probe	5’F-CAAGGTCGCAAGATTAGTGATGATGAACCAGG-3‘T
lin+circHPRT1 (*ex2-3*)	5’-primer	5’-GGAAAGGGTGTTTATTCCTCATGG-3’
NM_000194	3’-primer	5’-GGCCTCCCATCTCCTTCATCA-3’
	probe	5’F-TGGACAGGACTGAACGTCTTGCTCGAGAT-3‘T
PES1	5’-primer	5’-GAGTTCATGCACTACATTATCGCTGC-3’
NM_014303	3’-primer	5’-CTGCCCCAGTACCTCGGCC-3’
	probe	5’F-TGCCCTGCGCAAGGTCTTCCTGTC-3‘T
SOX2	5’-primer	5’-CCAGCGCATGGACAGTTACG-3’
NM_003106	3’-primer	5’-CTGGTCCTGCATCATGCTGTAG-3’
	probe	5’F-TGAACGGCTGGAGCAACGGCA-3‘T
cMAF	5’-primer	5’-CAGCGACAACCCGTCCTCTC-3’
NM_005360	3’-primer	5’-GGCTTCCAAAATGTGGCGTAT-3’
	probe	5’F-TAACTGAGCCCACTCGCAAGTTGGAGC-3‘T

probes: 5‘F-= 5‘FAMRA-; -3‘T = -3’TAMRA

Supplementary Table 10 gRNA sequences and PCR primers for validation.

**Name**	**gRNA/ primer**	**Sequence**
gRNA 5’ *ANRIL* exon 5	gRNA	5’-GCAGAGAAATACATTGAATA-3’
gRNA 3’ *ANRIL* exon 20	gRNA	5’-GTTAGTTTGGTTGAGGTAAG-3’
Ex1 PCR	5’-primer	5’-CGCGGATTCTGGTGCTGCTC-3’
	3’-primer	5’-GTCCAGATGTCGCGTCAGAGG-3’
Ex7 PCR	5’-primer	5’-CAGACTGGTCTTGAACTCTCGACCTC-3’
	3’-primer	5’-GCATTCAGGCCAAGGAAGAGAAG-3’
Ex5-20 PCR	5’-primer	5’-GGCAGGAGGATCTGGGGCATATG-3’
	3’-primer	5’-GAGCAAAGCATCAGGATCCTTAAAAG-3’

Supplementary Table 11 Antibodies for Western blotting (WB), native gel electrophoresis (NG), RNA-immunoprecipitation (RIP), immunofluorescent (IF) stainings and secondary antibodies.

Antibody	Supplier	Catalogue number	Primary/ secondary antibody	Host	Mono- /poly- clonal	WB	NG	RIP	IF
GAPDH	Fitzgerald	10R-G109a	p	mouse	m	+			
LAMIN B2	Santa Cruz	sc-377379	p	mouse	m	+			
PES1	Abcam	ab72539	p	rabbit	p	+	+		+
PES1	Santa Cruz	sc-166300	p	mouse	m	+			
TP53 (p53)	Abcam	ab26	p	mouse	m	+			
AGO2	Thermo Scientific	MA5-14861	p	rabbit	m			+	
BOP1	Santa Cruz	sc-390672	p	mouse	m	+	+	+	+
BRIX1	Santa Cruz	sc-373680	p	mouse	m			+	
NOLC1	Santa Cruz	sc-374033	p	mouse	m			+	
NOP14	Abnova	H00008602 -B01P	p	mouse	p			+	
PES1	Santa Cruz	sc-166300	p	mouse	m			+	
TP53 (p53)	Abcam	ab90363	p	mouse	m				+
Peroxidase labelled anti- mouse	Vektor Laboratories	PI-2000	s	horse	p	+			
Peroxidase labelled anti-rabbit	Vektor Laboratories	PI-1000	s	goat	p	+			
Alexa Fluor 568 anti-rabbit	Life Techno- logies	A-11036	s	goat	p				+
Alexa Fluor 568 anti-mouse	Life Techno- logies	A-11031	s	goat	p				+
